# Registered Nurses' and nursing students' perspectives on moral distress and its effects: A mixed‐methods systematic review and thematic synthesis

**DOI:** 10.1002/nop2.1913

**Published:** 2023-07-17

**Authors:** Tessa Watts, Anna Sydor, Dean Whybrow, Eunice Temeng, Rachael Hewitt, Rachael Pattinson, Christine Bundy, Richard G. Kyle, Bethan Jones

**Affiliations:** ^1^ School of Healthcare Sciences Cardiff University Cardiff UK; ^2^ Public Health Wales Cardiff UK; ^3^ Academy of Nursing University of Exeter Exeter UK; ^4^ School of Health and Social Wellbeing University of West of England Bristol UK

**Keywords:** mixed‐methods, moral distress, nursing students, nursing workforce, Registered Nurses, systematic review

## Abstract

**Aim:**

To examine Registered Nurses (RNs') and nursing students' perspectives on factors contributing to moral distress and the effects on their health, well‐being and professional and career intentions.

**Design:**

Joanna Briggs Institute mixed‐methods systematic review and thematic synthesis. Registered in Prospero (Redacted).

**Methods:**

Five databases were searched on 5 May 2021 for studies published in English since January 2010. Methodological quality assessment was conducted in parallel with data extraction.

**Results:**

Searches yielded 2343 hits. Seventy‐seven articles were included. Most were correlational design and used convenience sampling. Studies were mainly from North America and Asia and situated in intensive and critical care settings. There were common, consistent sources of moral distress across continents, specialities and settings. Factors related to perceived inability or failure to enact moral agency and responsibility in moral events at individual, team and structural levels generated distress. Moral distress had a negative effect on RNs health and psychological well‐being.

**Patient or Public Contribution:**

No patient or public contribution to this systematic review.

## 1 BACKGROUND

Global concern about the complex phenomenon of moral distress within nursing has been expressed for almost four decades. Morally challenging situations are common in healthcare and moral distress is not unique to nursing. However, experiencing moral distress is known to have profound personal and professional effects on individuals. It undermines integrity, functional competency and negatively impacts mood and intentions to remain in the workforce (Colville et al., 2019).

Initially coined by Jameton (1984), moral distress is an elusive concept which, in the context of nursing, evades conceptual clarity (Johnstone & Hutchinson, 2015; Pauly et al., 2012) and consensual definition (Deschenes et al., 2020; Morley et al., 2019). Indeed, Morley et al. (2019) found 20 moral distress definitions and identified five moral distress subtypes: moral constraint, moral conflict, moral tension, moral uncertainty and moral dilemmas (Morley, Bradbury‐Jones, & Ives, 2020). For this review, moral distress was defined as an ‘umbrella concept that describes the psychological, emotional and physiological suffering that may be experienced when we act in ways that are inconsistent with deeply held ethical values, principles or moral commitments’ (McCarthy & Deady, 2008, p. 1).

Nursing work is inherently demanding (Broetje et al., 2020). Higher rates of mental ill‐health among nurses compared with the general working population have been identified (Kinman et al., 2020; Martín‐Del‐Río et al., 2018). The risk of psychological morbidity among nurses is well documented (Chana et al., 2015; Chin et al., 2019; House of Commons, 2021; Melnyk, 2020; Melnyk et al., 2018). Evidence shows that nurses' stress is compounded by structural, organisational, workplace‐based challenges, including excessive and intensive workloads, staff shortages, difficult working conditions, shift work, incivility, team conflict, quality of leadership and management practices (Hartin et al., 2018, 2020; Lee & Kim, 2020; Tahghighi et al., 2017). The cumulative effect of repeated exposure to workplace stressors impacts on mental health (Stelnicki & Carleton, 2021), influencing staff turnover and decisions to leave the profession (Nursing and Midwifery Council, 2020; Royal College of Nursing, 2019), thereby contributing to the global nursing workforce retention crisis. Sustaining and retaining a healthy, motivated and appropriately supported nursing workforce is central to high quality, safe and effective care which optimises patient outcomes, reduces ‘missed care’ and preventable mortality, and meets population health needs (Aiken et al., 2014; Griffiths et al., 2018).

Moral distress is a key determinant of nurses' poor psychological and physical health. However, despite the ubiquity of morally challenging experiences in nursing practice, the influencing factors and effects of moral distress among nurses are poorly understood. This hampers the provision of appropriate organisational support, especially in the context of SARS‐Cov‐2 recovery, and the development of accessible interventions to mitigate the psychological effects of moral distress. To support and retain a healthy nursing workforce and inform planning for future public health emergencies, including pandemics, learning from the existing literature on moral distress with Registered and student Nurses before the SARS‐CoV‐2 pandemic is essential. This mixed‐methods systematic review examines RNs' and nursing students' perspectives on factors contributing to moral distress and the effects on their health, well‐being and professional and career intentions by answering the following review questions:

1. What factors contribute to moral distress among RNs and nursing students?
2. What are the effects of moral distress on their:
  - Health and well‐being?
  - Professional and career intentions?

## 2 METHODS

The systematic review was informed by the Joanna Briggs Institute (JBI) mixed‐methods systematic reviews methodology (MMSR) (Lizarondo et al., 2020) and registered in PROSPERO (CRD42021245362). The review is reported in accordance with the Preferred Reporting Items for Systematic Review and Meta‐Analysis (PRISMA) guidelines (Page et al., 2021).

### 2.1 Inclusion and exclusion criteria

Primary qualitative, quantitative and mixed‐methods research studies which focused on moral distress in Registered Nurses (RNs), nursing associates/apprentices/students working in healthcare settings and were published in English were included. Non‐empirical, opinion pieces, theoretical and methodological articles, reviews and editorials were excluded. Research studies were excluded if they were based on secondary data analysis, conducted in neonatal and social care settings, reported on healthcare professionals' moral distress where data were pooled for analysis, or did not meet any of the four quality criteria during the quality appraisal process, as detailed below.

### 2.2 Search strategy, study selection and data extraction

The search strategy was developed and tested in collaboration with a specialist health service systematic review librarian (EG). On 5th May 2021, one reviewer (ET) systematically searched the electronic databases MEDLINE, PsycINFO (via OvidSp), CINAHL (via EBSCO host), Embase (via Elsevier) and the Web of Science for studies published in English since 2010. This review was commissioned in the early stages of the SARS‐CoV‐2 pandemic. Given our timescales, the decision to run the searches between 2010 and 2021 was pragmatic, and taken in consultation with information specialists to ensure relative stability in the healthcare context within which nurses were working and experiencing moral distress. A combination of Medical Subject Headings (MeSH) search terms was used including moral*, distress, suffering, injury, residue, psychological distress, nurse and nurses. To enhance the sensitivity and refine the searches, Boolean operators (OR and AND) were used. A detailed description of the search strategies used in each database is presented in the online supplementary material (File S1). All hits were entered into EndNote and duplicates removed. Remaining hits were imported to Covidence SR management software. Additional duplicates were identified and removed.

All project team members were involved in the screening and selection process. Standardised systematic review methods (Centre for Reviews & Dissemination, 2009) were used. Firstly, two reviewers independently screened returned titles and abstracts, sifting these into a ‘yes’, ‘no’ or ‘maybe’ category. Where a definite decision based on title and abstract alone could not be made, the full text was retrieved and assessed. Secondly, full text of all potentially relevant abstracts were retrieved and independently assessed for inclusion by reviewers against the purposively designed eligibility criteria. Uncertainties for both first‐ and second‐level screening were resolved by the two reviewers. In the event of disagreement, an independent reviewer would arbitrate. However, arbitration was not required. Reasons for exclusion at full text review were recorded.

Data were extracted systematically using an adapted JBI mixed‐methods data extraction form and Covidence software. A second reviewer independently cross‐checked all data extraction forms for accuracy, integrity and completeness. To establish concordance, a third reviewer independently moderated a sample (10%) of extracted data. Extracted data included the author(s), year and country of publication, study aim and design, setting, number and characteristics of participants, approaches to sampling, data collection, analysis and quality appraisal outcome. In preparation for analysis and to facilitate the comparison and contrast of study findings systematically and coherently, for each study, a brief, textual, narrative summary reporting key findings relevant to the review questions was written.

### 2.3 Quality appraisal

Two reviewers independently assessed the quality of included studies using the Mixed Methods Appraisal Tool (MMAT) version 18 (Hong et al., 2018). The MMAT was constructed specifically for quality appraisal in mixed studies reviews (Hong et al., 2018; Pace et al., 2012). Each study was assigned a score based on the number of criteria met (25%—one criterion met; 100%—all criteria met). Studies were excluded if they met none of the quality criteria.

### 2.4 Data analysis and synthesis

Findings from qualitative, quantitative and mixed‐methods studies were synthesised thematically to address the review questions. The textual narrative summaries created during data extraction were aggregated and checked (TW). Guided by Thomas and Harden's (2008) approach to thematic synthesis, two researchers (TW, BJ) read and reread the aggregated textual summaries and corresponding articles. Initial, descriptive inductive codes were generated independently. Patterns within and between the studies were identified and following consultation with other team members for rigour.

## 3 RESULTS

### 3.1 Search results and overview of studies selected

**FIGURE 1:**
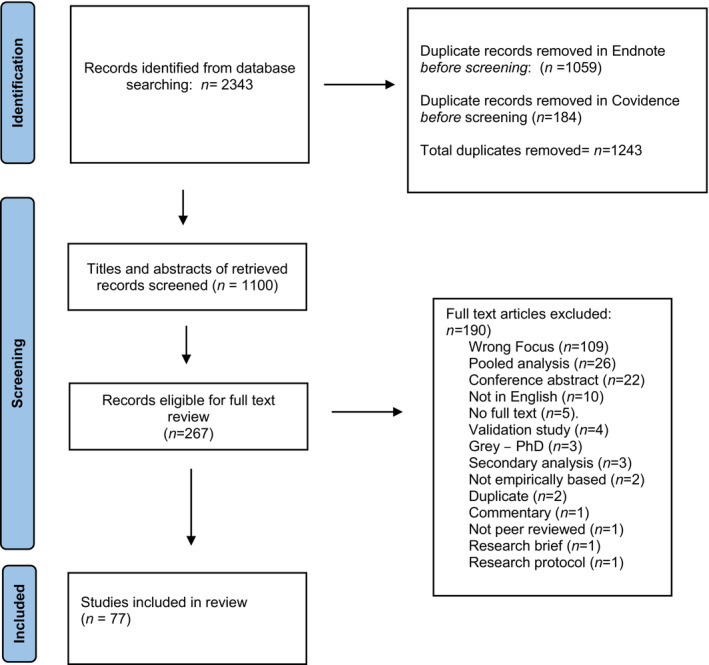
PRISMA 2020 flow diagram for new systematic reviews which included searches of databases and registers only. Adapted from: Page et al. (2021).

Figure 1 shows a PRISMA flow chart of search results. Following first‐ and second‐level screening, 77 articles (3.3%) were deemed suitable for inclusion.

Seventy‐seven articles published between 2010 and 2021 were included: 42 quantitative, 29 qualitative and six mixed‐methods studies. A synopsis of study characteristics are provided in Tables 1, 2, 3. Detailed summaries of these articles and the quality appraisal outcomes are provided in the online supplementary file (File S2).

**TABLE 1:** Synopsis of included quantitative studies.

Study author (year)	Country	Study design	Study setting	Study participants	Sample	Moral distress data collection measure
Abdolmaleki et al. (2019)	Iran	Cross‐sectional survey	Hospital: Emergency departments	Emergency department nurses	173	Hamric et al. (2012) Moral Distress Scale‐Revised
Ajoudani et al. (2019)	Iran	Cross‐sectional survey	Teaching hospitals	Nurses	278	Hamric et al. (2012) Moral Distress Scale‐Revised
Alberto Fruet et al. (2019)	Brazil	Cross‐sectional survey	Hospital: Haematology‐Oncology	Nurses, nursing technicians and nursing assistants	46	Moral Distress Scale (MDS) – Brazilian Version
Altaker et al. (2018)	United States	Cross‐sectional survey	Hospital: Intensive care	Intensive care nurses	238	Hamric et al. (2012) Moral Distress Scale‐Revised
Asayesh et al. (2018)	Iran	Cross‐sectional survey	Hospital: Intensive care	Intensive care nurses	117	Hamric et al. (2012) Moral Distress Scale‐Revised
Asgari et al. (2019)	Iran	Cross‐sectional survey	Social security hospitals	Critical care nurses	142	Hamric et al. (2012) Moral Distress Scale‐Revised
Bayat et al. (2019)	Iran	Cross‐sectional survey	Hospitals	Nurses	300	Corley et al.'s (2001) Moral Distress Scale
Berhie et al. (2020)	Ethiopia	Cross‐sectional survey	Regional state referral hospital	Nurses	412	Hamric et al. (2012) Moral Distress Scale‐Revised.
Borhani et al. (2014)	Iran	Cross‐sectional survey	Teaching hospital	Nurses	220	Corley et al.'s (2001) Moral Distress Scale
Browning (2013)	United States	Cross‐sectional survey	Hospital: Critical care units	Critical care nurses	277	Corley et al.'s (2001) Moral Distress Scale
Christodoulou‐Fella et al. (2017)	Cyprus	Cross‐sectional survey	Psychiatric care	Psychiatric nurses	206	Hamric et al. (2012) Moral Distress Scale‐Revised
Davis et al. (2012)	United States	Cross‐sectional survey	Hospital	Registered Nurses	1114	Hamric et al. (2012) Moral Distress Scale‐Revised
Delfrate et al. (2018)	Italy	Cross‐sectional survey	Hospital	Registered psychiatric nurses	228	Canciani et al.'s (2016) Moral Distress Scale for Psychiatric Nurses (Italian, revised)
Dodek et al. (2019)	Canada	Cross‐sectional survey	Hospital: Intensive care units	Intensive care nurses	428	Hamric et al. (2012) Moral Distress Scale‐Revised
Dyo et al. (2016)	United States	Cross‐sectional survey	Hospital	Registered Nurses	279	Corley et al.'s (2001) Moral Distress Scale
Emmamally and Chiyangwa (2020)	South Africa	Cross‐sectional survey	Private Hospital	Critical care nurses	74	Hamric et al. (2012) Moral Distress Scale‐Revised.
Evanovich Zavotsky and Chan (2016)	United States	Cross‐sectional survey	Hospital: Emergency department	Emergency Department nurses	198	Hamric et al. (2012) Moral Distress Scale‐Revised.
Fard et al. (2020)	Iran	Cross‐sectional survey.	Public and private hospitals	Nurses	150	Corley et al.'s (2001) Moral Distress Scale
Fernandez‐Parsons et al. (2013)	United States	Cross‐sectional survey	Hospital: Emergency department	Emergency Department Registered Nurses	51	Hamric et al. (2012) Moral Distress Scale‐Revised.
Ganz et al. (2013)	Israel	Cross‐sectional survey	Hospital: Intensive care units	Intensive care nurses	291	Corley et al.'s (2001) Moral Distress Scale
Haghighinezhad et al. (2019)	Iran	Cross‐sectional survey	Hospital: Intensive care units	Intensive care nurses	284	Iranian ICU Nurses' Moral Distress Scale
Hamaideh (2014) N = 130	Jordan	Cross‐sectional survey	Hospitals and clinics	Mental health nurses	130	Moral Distress Scale for Psychiatric Nurses
Harorani et al. (2019)	Iran	Cross‐sectional survey	Hospital: ICU, cardiac care and dialysis units	Nurses	300	Corley et al.'s (2001) Moral Distress Scale
Hatamizadeh et al. (2019)	Iran	Cross‐sectional survey	Hospital	Nurses	276	Corley et al.'s (2001) Moral Distress Scale
Hiler et al. (2018)	United States	Cross‐sectional survey	Hospitals: Critical care units	Critical care nurses	328	Hamric et al. (2012) Moral Distress Scale‐Revised.
Hou et al. (2021)	China	Cross‐sectional survey	Public hospitals: emergency department	Emergency department nurses	291	Sun et al.'s (2012) Chinese Version of Moral Distress Scale‐Revised (MDS‐R)
Karanikola et al. (2014) Italy	Italy	Cross‐sectional survey	International nursing conference	Intensive care nurses	556	Corley et al.'s (2001) Moral Distress Scale
Krautscheid et al. (2020)	United States	Pilot, cross‐sectional survey	University	Senior nursing students	60	Wocial and Weaver's (2013) moral distress thermometer (MDT)
Latimer et al. (2021)	United States	Pilot, cross‐sectional survey	Hospital	Ventricular assistance device (VAD) coordinators (nurses)	36	Measure of Moral Distress for Health Professionals (Epstein et al., 2019).
Laurs et al. (2020)	Lithuania	Cross‐sectional survey	Municipal hospitals	Registered Nurses	612	Hamric et al. (2012) Moral Distress Scale‐Revised.
Moaddaby et al. (2021)	Iran	Cross‐sectional survey	Hospitals: Intensive care units	Intensive care nurses	155	Corley et al.'s (2001) Moral Distress Scale
O'Connell (2015)	United States	Cross‐sectional survey	Internet nursing community	Critical care nurses	31	Hamric et al. (2012) Moral Distress Scale‐Revised
Pergert et al. (2019)	Sweden	Cross‐sectional survey	Hospital: oncology department	Paediatric nurses	278	Swedish Moral Distress Scale‐Revised
Rathert et al. (2016)	United States	Cross‐sectional survey	Trauma hospitals	Trauma nurses	290	Study‐specific and included one item about moral distress
Robaee et al. (2018)	Iran	Cross‐sectional survey	Hospital	Nurses	110	The ICU nurses' Moral Distress Scale
Sarkoohijabalbarezi et al. (2017)	Iran	Cross‐sectional survey	Hospital	Paediatric nurses	120	Hamric et al. (2012) Moral Distress Scale‐Revised.
Soleimani et al. (2019)	Iran	Cross‐sectional survey	Hospital	Nurses	193	Hamric et al. (2012) Moral Distress Scale‐Revised
Trautmann et al. (2015)	United States	Cross‐sectional survey	Hospital: Emergency department	Emergency department nurse practitioners	207	Hamric et al. (2012) Moral Distress Scale‐Revised.
Wands (2018)	United States	Cross‐sectional survey	Hospital	Registered Nurse anaesthetists	134	Ethical Stress Scale (ESS)
Wilson et al. (2013)	United States	Cross‐sectional survey	Hospital: Critical care nurses	Critical care nurses	105	Corley et al.'s (2001) Moral Distress Scale
Woods et al. (2015)	New Zealand	Cross‐sectional survey	Hospital	Registered Nurses	412	Hamric et al. (2012) Moral Distress Scale‐Revised
Yeganeh et al. (2019)	Iran	Cross‐sectional survey	Hospital: Intensive care	Intensive care nurses	180	Corley et al.'s (2001) Moral Distress Scale

**TABLE 2:** Synopsis of included qualitative studies.

Study author and year country	Country	Study design	Study setting	Participants	Sample size	Data collection
Atashzadeh Shorideh et al. (2012)	Iran	Qualitative descriptive research	Hospital	Intensive care nurses and educators	31	Individual interviews
Caram et al. (2019)	Brazil	Qualitative descriptive research	Hospital: Intensive care and surgical units	Intensive care and surgical nurses	13	Observation and semi‐structured interviews
Choe et al. (2015)	South Korea	Phenomenology	Hospital: Critical care setting	Critical care nurses	14	In‐depth, individual interviews
Crespo Drago et al. (2020)	Brazil	Qualitative descriptive research	Hospital	Nurse managers	17	Individual interviews and comic book completion
Deady and McCarthy (2010)	Ireland	Qualitative descriptive research	Acute psychiatric wards	Registered psychiatric nurses	8	Individual interviews
De Brasi et al. (2021)	Italy	Phenomenology	Hospital	Onco‐haematology nurses	28	Individual interviews
de Sousa Vilela et al. (2021)	Brazil	Qualitative descriptive research	Hospital: Intensive care	Intensive care nurses	12	Individual interviews
Escolar Chua and Magpantay (2019)	Philippines	Qualitative descriptive research	University	Senior nursing students	14	Individual interviews
Forozeiya et al. (2019)	Canada	Qualitative descriptive research	Hospital: Intensive care	Intensive care nurses	7	Individual interviews
Harrowing and Mill (2010)	Uganda	Ethnographic study	Referral centre	Nurses	24	Ethnographic methods
Hsun‐Kuei et al. (2018)	Taiwan	Grounded theory	Teaching hospitals	Staff nurses	25	Interviews
Jansen et al. (2020)	Norway	Qualitative descriptive research	Hospitals	Registered Psychiatric nurse specialists	16	Individual interviews
Ko et al. (2019)	Taiwan	Phenomenology	Hospital	Nurses	32	Individual interviews
Maluwa et al. (2012)	Malawi	Qualitative descriptive research	Various settings in one region of Malawi	Nurses	20	Individual interviews
Musto and Schreiber (2012)	Canada	Grounded Theory	Inpatient and community care	Registered Nurses and registered psychiatric nurses	12	Individual interviews
Nikbakht Nasrabadi et al. (2018)	Iran	Phenomenology	Hospital	Nurse managers	14	Individual interviews
Pavlish et al. (2016)	United States	Qualitative descriptive research	Association of nurse leaders	Nurse leaders	100	Critical incident narratives
Porr et al. (2019)	Canada	Grounded theory	Community	Registered Nurses	24	Individual interviews
Prestia et al. (2017)	United States	Qualitative exploratory study	Various states across the United States	Chief Nursing Officers	20	Individual interviews
Reader (2015)	United States	Narrative research	University	Nursing students	15	Individual interviews
Renno et al. (2018)	Brazil	Qualitative descriptive research	University	Undergraduate nursing students	58	Focus group interviews
Rezaee et al. (2019)	Iran	Qualitative descriptive research	Hospitals	Oncology nurses	25	Individual interviews
Ritchie et al. (2018)	Canada	Qualitative descriptive research	Urban setting in Western Canada	Nurse practitioners	6	Individual interviews
Robinson and Stinson (2016)	United States	Phenomenology	Hospital: Emergency department	Emergency department nurses	8	Individual interviews
Silverman et al. (2021)	United States	Qualitative descriptive research	Hospital	Acute care nurses	31	Individual interviews and focus groups
Wiegand and Funk (2012)	United States	Qualitative descriptive study	Hospital	Critical care nurses	47	Open‐ended survey
Wojtowicz et al. (2014)	Canada	Naturalistic Enquiry	University	Nursing students	7	Individual interviews
Wolf et al. (2016)	United States	Qualitative exploratory research	Conference	Emergency department nurses	17	Focus groups
Woods (2020)	New Zealand	Qualitative descriptive research	Hospital	Nurses	140	Qualitative survey

**TABLE 3:** Synopsis of included mixed‐methods studies.

Study author and year	Country	Design	Setting	Participants	Sample	Data collection
Langley et al. (2015)	South Africa	Mixed‐methods	Hospital: Intensive care	Intensive care nurses	65	Bespoke survey Focus groups (*n =* 4)
Mehlis et al. (2018)	Germany	Prospective mixed‐methods	Hospital	Oncologists and oncology nurses	89 (50 nurses)	Survey
Krautscheid et al. (2017)	United States	Mixed‐methods	University	Senior nursing students	267	Survey Demographic data Moral Distress Thermometer and a written narrative describing clinical situations contributing to moral distress
Prompahakul et al. (2021)	Thailand	Mixed‐methods	Hospital	General nurses	472 (survey) 20 (Interviews)	Survey Interviews
Sauerland et al. (2014)	United States	Mixed‐methods	Hospital	Acute and critical care Registered Nurses	225	Survey with open‐ended questions
Varcoe et al. (2012)	Canada	Mixed‐methods	Hospital	Nurses	292	Survey with open‐ended questions

#### 3.1.1 Study characteristics

All quantitative studies (Table 1) were cross‐sectional surveys and most used not only validated outcome measures, primarily Hamric et al.'s (2012) Moral Distress Scale‐Revised (MSD‐R) (n=), but also translated versions of the original English language MDS (Corley et al., 2001) or the MDS‐R. Most qualitative studies (*n =* 29) (Table 2) used a qualitative descriptive approach (*n =* 18). Five studies used phenomenology, while other approaches included grounded theory (*n =* 3), critical ethnography (*n =* 1), narrative (*n =* 1) and naturalistic enquiry (*n* = 1). A synopsis of mixed‐methods studies (*n* = 6) is presented in Table 3.

#### 3.1.2 Study populations

The majority of included studies (*n* = 77) were conducted in North America (*n* = 30): United States [*n* = 23] and Canada [*n* = 7]; Asia: (*n* = 26): Iran [*n* = 18]; China; Israel; Jordan; South Korea; Philippines; Taiwan [*n* = 2] and Thailand; Europe (*n* = 9): Norway; Sweden, Ireland; Italy [*n* = 3]; Cyprus; Germany and Lithuania. Other studies were from South America, specifically Brazil (*n* = 5); Africa (*n* = 5): Ethiopia; South Africa [*n* = 2]; Uganda and Malawi and Australasia: New Zealand [*n* = 2].

Most studies (*n* = 62) focused on moral distress among nurses in hospital settings specifically: intensive care (*n* = 14), critical care (*n* = 8), emergency departments (*n* = 7), haematology and oncology units (*n =* 5) and psychiatric units (*n =* 6). Six studies were conducted in universities with nursing students.

#### 3.1.3 Study quality

Eight of the 29 qualitative studies (28%) and seven of the 42 quantitative studies (17%) fulfilled all four MMAT quality criteria. None of the mixed‐methods studies fulfilled all MMAT quality criteria.

#### 3.1.4 Thematic synthesis

Moral distress was intrinsically connected to nurses and nursing students' perceived inability to act ethically, appropriately and simultaneously preserve the nursing identity and epistemology of person‐centred care and uphold core professional values, notably those relating to human dignity and advocacy (Alberto Fruet et al., 2019; Caram et al., 2019; Choe et al., 2015; de Sousa Vilela et al., 2021; Deady & McCarthy, 2010; Escolar Chua & Magpantay, 2019; Forozeiya et al., 2019; Harrowing & Mill, 2010; Hsun‐Kuei et al., 2018; Krautscheid et al., 2017; Mehlis et al., 2018; Prompahakul et al., 2021; Ritchie et al., 2018; Robinson & Stinson, 2016; Silverman et al., 2021; Wojtowicz et al., 2014; Wolf et al., 2016). For the first review question, three synthesised findings reflected factors contributing to nurses' moral distress: ‘*What can we do?*’: the pervading influence of individuals' characteristics; ‘*Nobody listens to you*’: relational dynamics and practices within intra‐ and interprofessional teams and ‘*The system is broken*’: the effect of structural constraints.

### 3.2 Factors contributing to moral distress among nurses

#### 3.2.1 ‘*What can we do?*’: The pervading influence of individuals' characteristics

A sense of powerlessness to intervene regarding care, treatment and decision‐making perceived as generating needless patient suffering and transgressing core professional values contributed to moral distress among RNs (Berhie et al., 2020; Crespo Drago et al., 2020; De Brasi et al., 2021; Deady & McCarthy, 2010; Harrowing & Mill, 2010; Ko et al., 2019; Langley et al., 2015; Nikbakht Nasrabadi et al., 2018; Prompahakul et al., 2021; Sauerland et al., 2014) and nursing students (Escolar Chua & Magpantay, 2019). This was invariably connected with interventions, treatment and care decisions perceived as futile (Asayesh et al., 2018; Browning, 2013; Choe et al., 2015; Ganz et al., 2013; Dodek et al., 2019; Dyo et al., 2016; Emmamally & Chiyangwa, 2020; Hiler et al., 2018; Hou et al., 2021; Karanikola et al., 2014; Ko et al., 2019; Latimer et al., 2021; Rezaee et al., 2019; Robinson & Stinson, 2016; Silverman et al., 2021; Wiegand & Funk, 2012; Wilson et al., 2013), overly aggressive (Rezaee et al., 2019; Wiegand & Funk, 2012) and inappropriate or unnecessary (Asgari et al., 2019; Browning, 2013; Choe et al., 2015; Christodoulou‐Fella et al., 2017; De Brasi et al., 2021; de Sousa Vilela et al., 2021; Fernandez‐Parsons et al., 2013; Forozeiya et al., 2019; Ganz et al., 2013; Ko et al., 2019; Laurs et al., 2020; Nikbakht Nasrabadi et al., 2018; Silverman et al., 2021) particularly, but not exclusively (de Sousa Vilela et al., 2021; Deady & McCarthy, 2010; Rezaee et al., 2019; Wojtowicz et al., 2014), in the context of end‐of‐life care.

We're with the patients a lot more than the providers … we see the futility a lot of the times, because we're like there's no way this person is going to make it out of here at the end, but the surgeons when they came in for ECMO, they're like keep going, keep going, keep going, keep going, never stop. (Silverman et al., 2021, p. 1147: United States, acute care)

Findings are mixed regarding how perceived professional autonomy to enact moral agency when faced with moral problems in practice connected with experiencing moral distress (Caram et al., 2019; Choe et al., 2015; Christodoulou‐Fella et al., 2017; Crespo Drago et al., 2020; Dodek et al., 2019; Karanikola et al., 2014; Sarkoohijabalbarezi et al., 2017; Yeganeh et al., 2019). However, RNs' (Deady & McCarthy, 2010; Harorani et al., 2019; Hsun‐Kuei et al., 2018; Ko et al., 2019; Pergert et al., 2019; Sauerland et al., 2014; Silverman et al., 2021; Varcoe et al., 2012) and nursing students' (Escolar Chua & Magpantay, 2019; Krautscheid et al., 2017; Renno et al., 2018) perceived lack of knowledge, self‐competence and confidence in their ability to articulate concerns and fulfil their perceived moral responsibilities in ethically challenging situations generated moral distress (Deady & McCarthy, 2010; Escolar Chua & Magpantay, 2019; Harorani et al., 2019; Hsun‐Kuei et al., 2018; Ko et al., 2019; Krautscheid et al., 2017; Pergert et al., 2019; Renno et al., 2018; Sauerland et al., 2014; Silverman et al., 2021; Varcoe et al., 2012).

While studies suggested that perceptions of moral distress might be influenced by sociodemographic factors, findings are conflicting and consistent correlation lacking. Some studies found no statistically significant correlation between age and perceived moral distress (Bayat et al., 2019; Dyo et al., 2016; Evanovich Zavotsky & Chan, 2016; Karanikola et al., 2014; Latimer et al., 2021; Mehlis et al., 2018; Prompahakul et al., 2021; Wilson et al., 2013). Others reported a significant, inverse correlation between age and moral distress (Abdolmaleki et al., 2019; Borhani et al., 2014; Christodoulou‐Fella et al., 2017; Emmamally & Chiyangwa, 2020; Ganz et al., 2013; Hamaideh, 2014; Hou et al., 2021; Laurs et al., 2020; Woods et al., 2015). That is, younger nurses experienced greater moral distress. A positive correlation between age and perceived moral distress intensity has also been identified (Browning, 2013; Moaddaby et al., 2021; O'Connell, 2015). Studies reporting the relationship between length of nursing service and perceived moral distress are inconsistent. Some studies (Alberto Fruet et al., 2019; Berhie et al., 2020; O'Connell, 2015) reported positive, occasionally significant (Alberto Fruet et al., 2019; Berhie et al., 2020) correlations. Others reported no statistically significant relationship (Bayat et al., 2019; Dyo et al., 2016; Emmamally & Chiyangwa, 2020; Evanovich Zavotsky & Chan, 2016; Karanikola et al., 2014; Latimer et al., 2021; Mehlis et al., 2018; Prompahakul et al., 2021; Wilson et al., 2013). An inverse correlation was reported in four studies (Borhani et al., 2014; Christodoulou‐Fella et al., 2017; Hamaideh, 2014; Latimer et al., 2021). Yet, the correlation was significant in just one study (Borhani et al., 2014). Various studies indicated a relationship between gender and perceived moral distress and suggested male and female nurses experience different levels of moral distress (Berhie et al., 2020; Borhani et al., 2014; Christodoulou‐Fella et al., 2017; Dyo et al., 2016; Emmamally & Chiyangwa, 2020; Rathert et al., 2016; Soleimani et al., 2019).

#### 3.2.2 ‘*Nobody listens*’: Relational dynamics and practices within intra and interprofessional teams

In morally challenging situations where patients' dignity, outcomes and optimal care were threatened and patient suffering occurred, colleagues' perceived ineptitude and unprofessional or unethical behaviours generated moral conflict. When unresolved, this contributed to moral distress among RNs (Asgari et al., 2019; Atashzadeh Shorideh et al., 2012; Choe et al., 2015; Christodoulou‐Fella et al., 2017; Emmamally & Chiyangwa, 2020; Hsun‐Kuei et al., 2018; Fernandez‐Parsons et al., 2013; Langley et al., 2015; Maluwa et al., 2012; Pergert et al., 2019; Prompahakul et al., 2021; Ritchie et al., 2018; Robaee et al., 2018; Sauerland et al., 2014; Silverman et al., 2021; Trautmann et al., 2015; Varcoe et al., 2012; Woods et al., 2015, Woods, 2020,) and nursing students (Escolar Chua & Magpantay, 2019; Krautscheid et al., 2017; Reader, 2015; Renno et al., 2018; Wojtowicz et al., 2014).

Some spoke up, directly asserted their clinical expertise to colleagues or informed their managers (Hsun‐Kuei et al., 2018; Nikbakht Nasrabadi et al., 2018; Prestia et al., 2017; Varcoe et al., 2012). Others, however, seemingly remained silent. This was primarily on account of interprofessional team hierarchies, notably the perceived enduring power of the medical profession (Atashzadeh Shorideh et al., 2012; Caram et al., 2019; de Sousa Vilela et al., 2021; Deady & McCarthy, 2010; Escolar Chua & Magpantay, 2019; Ko et al., 2019; Langley et al., 2015; Pavlish et al., 2016; Renno et al., 2018; Silverman et al., 2021; Wolf et al., 2016), encapsulated in the following data extract:

Physicians believed [sic] they are above us. They order for patients and they expect us to obey them and not tell them about wrong orders. We are obliged to carry out their orders without asking any question. (Atashzadeh Shorideh et al., 2012, p. 471: Iran, intensive care)

RNs and nursing students perceived that they were subordinate (Atashzadeh Shorideh et al., 2012; Krautscheid et al., 2017), powerless (Deady & McCarthy, 2010), invisible (de Sousa Vilela et al., 2021) and their role, unique insights and contribution to care undervalued (Atashzadeh Shorideh et al., 2012; Caram et al., 2019; de Sousa Vilela et al., 2021; Deady & McCarthy, 2010; Hsun‐Kuei et al., 2018; Maluwa et al., 2012; Ritchie et al., 2018; Varcoe et al., 2012; Wolf et al., 2016).

The physician does not assess the patient, does not do a physical exam. The entire assessment of the patient is done by the nurses, it is the nurses who pass on the information. And even with our concern, they do not value our knowledge at all. (de Sousa Vilela et al., 2021, p. 5: Brazil, intensive care)

Fear of negative repercussions (Atashzadeh Shorideh et al., 2012; Prompahakul et al., 2021) and alienation (Deady & McCarthy, 2010), unsupportive, ineffective managers (Atashzadeh Shorideh et al., 2012; Caram et al., 2019; Hsun‐Kuei et al., 2018; Langley et al., 2015; Varcoe et al., 2012; Wolf et al., 2016; Woods, 2020) and a desire to avoid team conflict were reported.

We're trained to vocalize our concerns and ask the hard questions and debate, but we're reprimanded for that by our managers. (Ritchie et al., 2018, p. 104: Canada, Continuing care)

What stops me from acting was I am part of a team, which should be cohesive. (Deady & McCarthy, 2010, p. 6: Ireland, Psychiatry)

RNs articulated that failing to speak up intensified their moral distress experience, particularly when care standards fell below their personal and professional practice standards, and they felt complicit in prolonging suffering (Deady & McCarthy, 2010). To mitigate moral distress in such circumstances, the importance of post‐incident team reflection was recognised (Deady & McCarthy, 2010). Yet, within and between teams, inadequate or insufficient communication, consultation and collaboration were identified as common problems compounding their moral distress (Atashzadeh Shorideh et al., 2012; De Brasi et al., 2021; de Sousa Vilela et al., 2021; Langley et al., 2015; Mehlis et al., 2018; Pavlish et al., 2016; Prompahakul et al., 2021; Rezaee et al., 2019; Ritchie et al., 2018). Furthermore, RNs who reported poor team communication were almost five times more likely to experience moral distress compared with those experiencing good team communication (Berhie et al., 2020).

#### 3.2.3 ‘A slave to the system’: The effect of structural constraints

The organisational environment contributed to RNs' experiences of moral distress. Within complex organisations, they recognised their role as conductors of care (Caram et al., 2019). However, there was scepticism that private sector, market‐driven institutional values and cultures privileged economic needs, managerialism, metrics and improving productivity over patients' needs and concerns.

It's all about the scores and the numbers. We're pulling them out of the rooms now and you're putting someone in the hallway who according to your policy should be on a monitor. (Wolf et al., 2016, p. 40: United States, emergency department)

Sometimes, a bed is free in the ICU, but if the patient depends on the public service, we pretend it is not free. I understand the economic aspect, because the institution needs money, but we [nurses] suffer because of it. (Caram et al., 2019, p. 6: Brazil, acute and intensive care)

Participants in one study (Choe et al., 2015) described situations where the inability to pay medical bills and thereby contribute to the institution's income meant homeless patients were discharged or transferred. Ritchie et al. (2018) found that institutional policy prohibited overtime working. Participants perceived this constrained professional practice and, impacted negatively on patients when timely responses were crucial to optimising outcomes.

RNs articulated that organisational expectations, policies and mandates, particularly those regarding managing bureaucracy and the flow of information, disregarded their core professional beliefs and values and impeded the accomplishment of their idealised role as direct care givers:

We do a lot of bureaucratic work. So, it seems that I am a ‘secretary with a degree’. I do not want this. (Caram et al., 2019, p. 4: Brazil, acute and intensive care)

This is our…choice between good care and good documentation. You [can be] a really good nurse on paper or you can actually be a really good nurse, but you don't have time to be both. (Wolf et al., 2016, p. 41: United States, emergency department)

Nursing students reported that their practice experiences, including witnessing outdated best practice (Renno et al., 2018) and being unsupported regarding their concerns did not live up to the view of nursing to which they were being socialised (Wojtowicz et al., 2014), and contributed to moral distress.

Privileging routinised, task‐orientated approaches to care (Caram et al., 2019; Choe et al., 2015; Rezaee et al., 2019; Silverman et al., 2021; Varcoe et al., 2012) in organisational environments of cost containment (Jansen et al., 2020; Pergert et al., 2019; Prestia et al., 2017; Ritchie et al., 2018), inadequate, unsafe nurse staffing ratios (Caram et al., 2019; Choe et al., 2015; Deady & McCarthy, 2010; Delfrate et al., 2018; Forozeiya et al., 2019; Hsun‐Kuei et al., 2018; Jansen et al., 2020; Maluwa et al., 2012; Pergert et al., 2019; Prestia et al., 2017; Rezaee et al., 2019; Silverman et al., 2021; Varcoe et al., 2012) and excessive, overwhelming workloads (Hsun‐Kuei et al., 2018; Silverman et al., 2021;Varcoe et al., 2012; Wolf et al., 2016), juxtaposed against high patient acuity and insufficient time correlated with reported perceptions of lower standards of care.

We usually have one or two patients max [*in the MICU*] And now, I have 6, 7, 8 patients, and they're all, like, most of them should be one‐to‐ones. (Silverman et al., 2021, p. 1150: United States, acute care)

There are many patients who need attention and you are all alone. There are a lot of activities to be carried out urgently but you find yourself not able to do them. As a result your patient suffers. (Maluwa et al., 2012, p. 199: Malawi, various settings)

Furthermore, reports of unreliable or insufficient essential equipment, for example, bed linen, personal protective equipment, thermometers, suction machines, catheters and medications, in low‐, middle‐ and high‐income countries, were documented (Atashzadeh Shorideh et al., 2012; Deady & McCarthy, 2010; Harrowing & Mill, 2010; Maluwa et al., 2012; Silverman et al., 2021; Wolf et al., 2016).

“The patient needed blood. There was a need to collect blood from a blood bank of another institution but there was no transport. Patient's condition deteriorated. I felt very bad.” (Maluwa et al., 2012, p. 200: Malawi, various settings)

Visible manifestations of the dominant organisational values and culture disrupted RNs' identity, generated moral conflict and moral tension and triggered moral distress (Choe et al., 2015; Deady & McCarthy, 2010; Maluwa et al., 2012; Prestia et al., 2017; Ritchie et al., 2018; Wolf et al., 2016; Woods, 2020).

### 3.3 ‘I'm totally overwhelmed’: The effects of moral distress on nurses

The moral distress derived from RNs' perceived inability to act in accordance with core professional values and optimise timely, safe, effective high‐quality person‐centred holistic care generated adverse biopsychosocial sequalae. Furthermore, findings from numerous studies indicated how the experience of frequent and intense moral distress impacted negatively on their professional intentions. By way of contrast, there were no reports of the effects of moral distress on nursing students in the six studies retrieved.

Physical manifestations of moral distress among RNs were reported in studies from Iran (Fard et al., 2020), Canada (Forozeiya et al., 2019), Norway (Jansen et al., 2020), USA (Prestia et al., 2017; Sauerland et al., 2014; Wilson et al., 2013; Wolf et al., 2016) and Uganda (Harrowing & Mill, 2010). Symptoms experienced included fatigue (Harrowing & Mill, 2010; Wolf et al., 2016), insomnia (Fard et al., 2020; Forozeiya et al., 2019; Jansen et al., 2020; Wilson et al., 2013; Wolf et al., 2016), hypertension (Jansen et al., 2020; Wolf et al., 2016) and appetite loss (Wolf et al., 2016).

My body's given up on eating, like I long since have not been hungry anymore. Then at the end of the night, when I [urinate], it's orange, and I think, ‘Oh my God, my kidneys are going to shut down.’ What we're doing to our bodies to take care of other people's bodies. (Wolf et al., 2016, p. 43: United States, emergency department)

Moral residue, the enduring, cumulative effect of morally distressing situations (Stovall et al., 2020), manifested in insomnia, cardiovascular, gastrointestinal and menstrual problems (Pavlish et al., 2016), alopecia (Sauerland et al., 2014) and activated exacerbations of physical and psychological illnesses (Pavlish et al., 2016).

Psychological effects of RNs' moral distress were reported in studies from Brazil (de Sousa Vilela et al., 2021), Canada (Forozeiya et al., 2019; Musto & Schreiber, 2012; Porr et al., 2019; Varcoe et al., 2012), Iran (Nikbakht Nasrabadi et al., 2018), Ireland (Deady & McCarthy, 2010), New Zealand (Woods, 2020), Norway (Jansen et al., 2020), Taiwan (Hsun‐Kuei et al., 2018), Uganda (Harrowing & Mill, 2010) and the United States (Prestia et al., 2017; Sauerland et al., 2014; Wiegand & Funk, 2012; Wilson et al., 2013; Wolf et al., 2016). Anger and frustration were not only responses to the moral distress generated by systemic constraints, notably workload (Varcoe et al., 2012; Wolf et al., 2016), but also a sense of powerlessness to act in accordance with professional values (de Sousa Vilela et al., 2021; Hsun‐Kuei et al., 2018; Wiegand & Funk, 2012; Wolf et al., 2016), make meaningful change (Musto & Schreiber, 2012; Varcoe et al., 2012) or discuss moral concerns (Deady & McCarthy, 2010).

I left here very distressed! It was a situation of a lot of conflict, anguish, frustration! I left frustrated because I didn't do what I could for the patient! I asked for intramuscular medication, but he [*doctor*] said she could wait for the procedure. So, I became nothing, because I spoke, the patient got worse and nothing was done. (de Sousa Vilela et al., 2021, p. 6: Brazil, intensive care)

RNs articulated that the moral distress associated with having insufficient time to spend with patients, episodes of ‘missed care’, and suboptimal care standards resulted in anxiety (Forozeiya et al., 2019; Nikbakht Nasrabadi et al., 2018; Porr et al., 2019; Varcoe et al., 2012), shame (Nikbakht Nasrabadi et al., 2018; Varcoe et al., 2012), guilt (Deady & McCarthy, 2010; Harrowing & Mill, 2010; Jansen et al., 2020; Porr et al., 2019; Wolf et al., 2016; Woods, 2020) and fear (Varcoe et al., 2012; Wolf et al., 2016). Many reported feeling low, despair, and finding less meaning in life as a result of moral distress (Harrowing & Mill, 2010; Jansen et al., 2020; Wiegand & Funk, 2012). Reported feelings of helplessness and hopelessness were not uncommon (Harrowing & Mill, 2010; Nikbakht Nasrabadi et al., 2018; Prestia et al., 2017; Wiegand & Funk, 2012). RNs experienced the weight of moral residue (Deady & McCarthy, 2010; Jansen et al., 2020; Porr et al., 2019; Prestia et al., 2017; Sauerland et al., 2014; Woods, 2020). This was manifested in loss of confidence in their nursing judgements and abilities (Jansen et al., 2020; Prestia et al., 2017; Sauerland et al., 2014), depression (Deady & McCarthy, 2010; Prestia et al., 2017) and feeling traumatised, paranoid (Prestia et al., 2017) and burnt‐out (Deady & McCarthy, 2010).

Many RNs articulated how their social relationships, networks and activities, and their work performance were adversely affected (Forozeiya et al., 2019; Jansen et al., 2020; Robinson & Stinson, 2016; Wilson et al., 2013).

It [*moral distress*] affects my family life, it affects my relationships, it affects my patients, and my relationships with my peers. (Robinson & Stinson, 2016, p. 238: United States: Emergency Department)

Some distanced themselves from loved ones and social activities (Forozeiya et al., 2019; Jansen et al., 2020; Robinson & Stinson, 2016). Others reported using unhelpful coping strategies including substance misuse, food or alcohol consumption (Evanovich Zavotsky & Chan, 2016; Robinson & Stinson, 2016; Wolf et al., 2016).

‘Oh my God, it's a 2‐martini night,’ or ‘Oh, I need to go home and have a glass of wine,’ and that gives me distress thinking okay now I'm thinking I'm turning to alcohol to calm this day I've had, which shouldn't ever be. (Wolf et al., 2016, p. 43)

Dreading the workplace (Forozeiya et al., 2019; Jansen et al., 2020), to protect themselves, some RNs reported distancing themselves from patients (Krautscheid et al., 2017; Robinson & Stinson, 2016; Varcoe et al., 2012) and the workplace (Forozeiya et al., 2019; Robinson & Stinson, 2016).

When you are experiencing this, you don't want to come to work. You try to distance yourself from your patients. You try to be cold and uncaring. (Robinson & Stinson, 2016, p. 238: United States, Emergency Department)

Moral distress meant some RNs contemplated working fewer hours (Forozeiya et al., 2019; Nikbakht Nasrabadi et al., 2018), taking a career break (Jansen et al., 2020) or leaving their workplace (Asayesh et al., 2018; Borhani et al., 2014; Christodoulou‐Fella et al., 2017; Davis et al., 2012; Evanovich Zavotsky & Chan, 2016; Fernandez‐Parsons et al., 2013; Forozeiya et al., 2019; Hou et al., 2021; Jansen et al., 2020; Nikbakht Nasrabadi et al., 2018; Robinson & Stinson, 2016; Wilson et al., 2013; Woods et al., 2015) or even the profession (Alberto Fruet et al., 2019). Studies indicated a connection, between more frequent and/or intense moral distress and the intention to leave a position (Dyo et al., 2016; Hamaideh, 2014; Hatamizadeh et al., 2019; Hou et al., 2021; Laurs et al., 2020; Prompahakul et al., 2021; Soleimani et al., 2019). Others reported having left their workplace or positions completely (Asayesh et al., 2018; Evanovich Zavotsky & Chan, 2016; Fernandez‐Parsons et al., 2013; Varcoe et al., 2012; Wilson et al., 2013) or transferred to work elsewhere due to moral distress (Deady & McCarthy, 2010; Varcoe et al., 2012).

However, not all RNs who had experienced moral distress left or considered leaving their positions (Borhani et al., 2014; Evanovich Zavotsky & Chan, 2016). Some used moral distress as a learning experience to drive them. For example, a subsection of participants in one study (Varcoe et al., 2012) reported that their moral distress motivated them and enabled them to build resolve. Nursing students experiencing moral distress reported seeing it as a form of learning, to avoid this happening to others in the future (Renno et al., 2018).

## 4 DISCUSSION

### 4.1 Understanding factors contributing to moral distress among RNs and nursing students

Evidence for the contribution of individual characteristics, including, age, length of service and gender, on moral distress was inconclusive. There is a need for further research to examine whether there are common individual characteristics that exacerbate nurses' experiences of moral distress. Identifying those who are most at risk of experiencing moral distress may enable more effective targeting and tailoring of interventions, as well as crucial learning around factors that might be protective against moral distress, especially among nurses working in similar roles and clinical environments. This evidence would be vital to inform development of interventions to prevent moral distress rather than mitigating the effects of moral distress that has already occurred and caused harm.

However, studies examining factors contributing to moral distress experiences were mostly correlational and used convenience sampling, which in itself runs the risk of selection bias. Furthermore, different measures were used to assess moral distress (Supplementary Material File S2 Table S1). Nevertheless, included studies mostly used established, validated outcome measures which focus on the frequency and intensity of moral distress across different items including, for example, end‐of‐life care, unsafe staffing, clinical decision‐making, institutional constraints, workplace culture and autonomy. Mainly these measures were the Moral Distress Scale‐Revised (MDS‐R) (*n =* 19) (Hamric et al., 2012), a scaled back version of Corley et al.'s (2001) seminal Moral Distress Scale (MDS) which, in this review, was used by 12 included studies. Three studies (Alberto Fruet et al., 2019; Hou et al., 2021; Pergert et al., 2019) used translated versions of the original English language MDS (Corley et al., 2001) and MDS‐R (Hamric et al., 2012), two used a version of the MDS adapted for psychiatry (Delfrate et al., 2018; Hamaideh, 2014) and one used Epstein et al.'s (2019) Measure of Moral Distress for Healthcare Professionals which is based on the MDS. However, measures used in the remaining five studies (Haghighinezhad et al., 2019; Krautscheid et al., 2020; Rathert et al., 2016; Robaee et al., 2018; Wands, 2018) were not underpinned by either the MDS or MDS‐R. Furthermore, Rathert et al. (2016) developed a bespoke measure focusing on ethical issues and conceptualised moral distress as moral stress. Notwithstanding the significance and immense contribution of Corley et al.'s (2001) seminal work in terms of enhancing our understanding of moral distress among nurses and for the purpose of research, arguably there is much more work to be done, not least because of the immense global societal change in the intervening years conjoined with serious concerns about the retention and sustainability of the nursing workforce worldwide. In addition to measures of moral distress, longitudinal assessment of how moral distress (and associated constructs including moral injury) develops is needed, as well as studies of the impact of interventions implemented to mitigate moral distress with long‐term follow‐up.

Despite equivocal evidence around the relationship between individual factors and moral distress, organisational factors, including RNs' and nursing students' perceived autonomy, ability to advocate and opportunity to raise concerns around care, were consistently reported to contribute to nurses' experiences of moral distress. Insufficient institutional support to behave ethically, inadequate resources, insufficient staffing and a wider ‘culture of silence’ (Pavlish et al., 2016) all precipitated moral distress. Yet, insufficient resources and poor staffing levels were triggered by high levels of moral distress among team members, creating a vicious cycle (Delfrate et al., 2018; Ganz et al., 2013; Harrowing & Mill, 2010; Hsun‐Kuei et al., 2018; Silverman et al., 2021).

This emphasises the need to respond to moral distress through preventative organisational strategies in addition to individually focussed interventions. Existing supportive interventions for tackling moral distress include Moral Distress Reflective Debriefs (Morley & Horsburgh, 2021) and the Moral Distress Debriefing Framework (Shashidhara & Kirk, 2020). Hence, cultivating organisational cultures that optimise staff support and open safe spaces for discussion of morally challenging experiences through, for example, clinical ethics services or effective, reflective and supportive clinical supervision should be prioritised (Dittborn et al., 2021; Morley, Sese, et al., 2020), especially in the wake of COVID‐19. Indeed, reporting findings from their recent study, Dittborn et al. (2021) showed how clinical ethics support services supported healthcare professionals in ethically challenging situations during the COVID‐19 pandemic. However, further robust empirical investigation of these interventions to ascertain potential impact on moral distress experienced is needed. Similarly, reviewing and promoting existing organisational policies that enable nurses to raise concerns, promote nurses' advocacy role and support effective intra‐ and inter‐professional working through the lens of mitigating moral distress could serve to avert and ameliorate the impacts of morally challenging situations. Given the ubiquity of moral challenge in healthcare practice, removal of moral complexity is an unattainable goal. However, a renewed policy focus may prevent onset of moral distress, moral injury and, in turn, the short‐ and long‐term harms on nurses' physical and psychological health.

### 4.2 Addressing the effect of moral distress on nurses' health, well‐being, professional and career intentions

Moral distress disrupted nurses' physical and psychological health, well‐being and professional and career intentions. Nurses reported experiencing physical symptoms of fatigue, insomnia, hypertension, appetite loss, and exacerbation of existing cardiovascular, gastrointestinal and menstrual problems. Psychological effects included anxiety, depression, anger, frustration, helplessness, hopelessness, shame, guilt and fear which negatively affected well‐being. Interventions to support nurses experiencing moral distress therefore need to recognise the diversity of symptoms and sequalae of moral distress and provide holistic, integrated physical and mental healthcare in response. Similarly, both the short‐ and longer‐term effects of experiencing moral distress identified in our systematic review need to be supported. For example, nurses described how their experience of moral distress left them feeling traumatised, shocked or haunted (Forozeiya et al., 2019; Harrowing & Mill, 2010; Varcoe et al., 2012). There is considerable risk that the moral distress experienced by nurses (and other healthcare professionals) during the SARS‐CoV‐2 pandemic will result in moral injury and increased prevalence of PTSD. Indeed, emerging international evidence has documented concerning levels of reported PTSD symptoms among nurses and other healthcare workers, particularly among those who worked on the SARS‐CoV‐2 pandemic frontline (Bae et al., 2022; Levi & Moss, 2022; Moon et al., 2021). Timely signposting and referral to specialist psychological support services therefore needs to be a core component of interventions developed to mitigate moral distress to support recovery, rebuilding and retention of the nursing workforce.

Moral distress was also associated with increased risk of workforce turnover and loss. Experiencing moral distress resulted in as many as a quarter of nurses considering leaving their current role and up to half intending to leave the nursing profession. Prior to the SARS‐CoV‐2 pandemic, the nursing workforce was already depleted, with a deficit of 6 million nurses globally (World Health Organization, 2020). Shortfalls are predicted to increase (Douglas et al., 2020) due to an ageing international nursing workforce (Denton et al., 2021; Kwok et al., 2016; Ryan et al., 2017). Demand for healthcare is intensifying due to changing patient demographics, widening health inequalities and increasing chronicity. There are serious implications for the quality and safety of care provision and the health and well‐being of the nursing workforce. Protecting, sustaining and retaining a healthy, motivated and appropriately supported nursing workforce is central to the delivery of high quality, safe and effective care and meeting current and future population health needs (Gray et al., 2020; World Health Organization, 2021). The risk of further loss of nursing personnel and expertise in the wake of the COVID‐19 due to moral distress pandemic places urgency on healthcare organisations and governments internationally to develop national strategies, organisational policies and interventions to mitigate the impact of moral distress on the nursing workforce.

The effects of moral distress on nursing students' own health, well‐being and intentions to remain do not appear to have been reported in the literature. Yet interestingly, nursing students responded to their moral distress by seeing it as a form of learning. They wanted to prevent this happening to others as they developed in their careers (Renno et al., 2018). This represents positive change from difficult situations: a form of post‐traumatic growth. Yet, there is an inherent risk that repeated exposure to moral distress may normalise it.

Our findings have implications for nursing education across all levels and preparing nursing students—the future workforce—for their clinical practice, including practice in a public health emergency. Nursing students are taught to manage their own resilience (Walsh et al., 2020) as they will become autonomous professionals and are expected to act ethically. Nurse education focuses on professional development and patients' interests and autonomy within the bounds of professional codes of conduct (e.g. Nursing and Midwifery Council, 2020). Arguably, this focus may lead to potential moral distress if the ability to exercise professional autonomy, act ethically to promote and uphold the patients' interests and remain resilient is obstructed by wider circumstance over which they have little control.

### 4.3 Strengths and limitations

Our systematic review was conducted by a multidisciplinary review team with a minimum of two reviewers engaged in the independent screening and extracting process. Some aspects of systematic review methodology were simplified to produce a review in a short enough time frame for the findings to remain relevant as healthcare services shift to the recovery phase of the pandemic. More specifically, searches were limited from 2010 to 2021 and empirical literature focused on nurses published in the English language. It is entirely possible that some potentially useful studies, notably those not published in the English language have been omitted. We also excluded pre‐prints and consequently identified only one study focusing on moral distress among nurses in the context of a pandemic. It is highly likely that over time the empirical literature pertaining to moral distress in the context of SARS‐CoV‐2 will grow. By limiting the search dates in this way we have ensured that the evidence assessed has context and relevance to current policy and practice.

## 5 CONCLUSIONS

This systematic review is important and timely given wider changes in the healthcare landscape and the SARS‐CoV‐2 pandemic which has substantially increased pressure on nurses and others providing care. This review adds specifically to understanding the effects of moral distress on RNs and nursing students. Several factors contribute to their moral distress experience that may be related to a perceived inability to enact moral agency. Experiences of moral distress are complex, relational and located at individual, team organisational and structural levels. The moral distress experience does not occur in a vacuum and there is potential for the interplay of complex relationships between individuals and organisational structures. Accordingly, moral distress is an inherently relational, complex and contextualised phenomenon. In challenging situations, there was a perception that RNs and nursing students were unable to enact an idealised version of their role. RNs and nursing students were constrained by personal perceptions of powerlessness, insufficient specialist practice and ethical knowledge, a perceived lack of agency to do the best for patients, and their families, and, at structural levels, relational and organisational constraints. Although encouraged to develop their own resilience, RNs and nursing students may be unable to exercise professional autonomy and uphold patient interests.

Moral distress impacted RNs' health and well‐being and manifest in emotional reactions including guilt, self‐doubt, loss of self‐confidence, anger and frustration. Health‐threatening behaviours were also identified. These emotions and behaviours may have detrimental longer term consequences for RNs. Enduring tropes of selfless and angelic nurses may further exacerbate the focus on the individual nurse, implying that the problem is a personal failing, lack of competence or transgression of professional codes. Increasing incidence of moral distress has implications for the nursing workforce. Specifically, a vicious cycle may develop in which RNs leave and those who continue are left under increasing pressure exacerbating moral distress in the workforce. The effects of moral distress on nursing students' own health, well‐being and intentions to remain does not appear to have been reported in the literature. Such research is urgently needed to sustain and protect the profession and optimise future patient safety.

## AUTHOR CONTRIBUTIONS

Tessa Watts, Richard G. Kyle and Christine Bundy contributed to the conceptualisation and design of the study. Eunice Temeng, Tessa Watts, Anna Sydor, Dean Whybrow, Rachael Hewitt and Rachael Pattinson were responsible for retrieving and assessing studies for inclusion in the review. Tessa Watts and Bethan Jones were responsible for thematic synthesis. Tessa Watts and Bethan Jones drafted the first version of the article. All authors critically reviewed the article and have read and approved the final article.

## FUNDING INFORMATION

This work was funded by Public Health Wales. Public Health Wales is an NHS organisation providing professionally independent public health advice and services to protect and improve the health and well‐being of the population of Wales. However, the views in this article are entirely those of the authors and should not be assumed to be the same as those of Public Health Wales.

## CONFLICT OF INTEREST STATEMENT

Professor Richard Kyle was employed by Public Health Wales when the review was commissioned.

## ETHICS STATEMENT

Research Ethics Committee approval was not required for this mixed‐methods systematic review.

## PATIENT CONSENT

Patient consent was not required for this mixed‐methods systematic review.

## Supporting information


File S1.

File S2.

## Data Availability

Data available in article supplementary material.
