# Supplementary material for: Registered Nurses' and nursing students' perspectives on moral distress and its effects: A mixed‐methods systematic review and thematic synthesis
Source: Nurs Open. 2023 Jul 17;10(9):6014–32. doi: 10.1002/nop2.1913 (PMC10416007; doi:10.1002/nop2.1913)
Supplement: Supplementary file 1 — File S1. [file NOP2-10-6014-s001.docx]

**Supplementary File S1: Table S1 MEDLINE SEARCH Strategy**

Interface: **EBSCO**

Database: **Medline**

Limiters: **2010/01/01-2021/04/30; English language**

Search modes: Find all my search terms: Boolean/Phrase; MeSH (MH)

| **ID** | **QUERY** | **Results** |
| --- | --- | --- |
| **1** | (MH "Morals+") | 172,532 |
| **2** | (MH "Psychological Distress") | 1,629 |
| **3** | #1 AND #2 | 30 |
| **4** | Moral* N2 (distress* OR suffering* OR stress* OR injur* OR concern* OR transgression* OR residue) | 2,675 |
| **5** | #3 OR #4 | 2,689 |
| **6** | (MH "Nurses+") | 90,139 |
| **7** | "Staff Nurses" | 20,356 |
| **8** | "Registered Nurses" | 9,564 |
| **9** | (MH "Students, Nursing") | 25,516 |
| **10** | (MH "Advanced Practice Nursing") | 1,770 |
| **11** | "Practical Nurses" | 862 |
| **12** | Nurse OR Nurses | 366,436 |
| **13** | #6 OR #7 OR #8 OR #9 OR #10 OR #11 OR #12 | 388,857 |
| **14** | #5 AND #13 | 776 |
| **15** | **#5 AND #13 Limiters applied** | **585** |

#### **Supplementary File S1: Table S2: Embase SEARCH Strategy**

Database: Ovid Embase

Limiters: **Published Date: 2010/01/01-2021/05/05; English language**

Search modes: Find all my search terms: **Boolean/Phrase; MeSH (MH)**

| ***ID*** | ***SEARCH QUERY*** | ***Results*** |
| --- | --- | --- |
| ***1*** | *(MH "Morals+")* | 42,981 |
| ***2*** | (MH "Psychological Distress") | 28,442 |
| ***3*** | #1 AND #2 | 83 |
| ***4*** | Moral* adj2 (distress* OR suffering* OR stress* OR injur* OR concern* OR transgression* OR residue) | 2,781 |
| ***5*** | #3 OR #4 | 2,827 |
| ***6*** | (MH "Nurses+") | 198,891 |
| ***7*** | (MH "Staff Nurses") | 1,275 |
| ***8*** | (MH "Registered Nurses") | 4,984 |
| ***9*** | (MH "Students, Nursing+") | 28,641 |
| ***10*** | (MH "Advanced Practice Nurses+") | 41,599 |
| ***11*** | (MH "Practical Nurses") | 225 |
| ***12*** | Nurse OR Nurses | 437,555 |
| ***13*** | #6 OR #7 OR #8 OR #9 OR #10 OR #11 OR #12 | 455,433 |
| ***14*** | #5 AND #13 | 865 |
| ***15*** | #5 AND #13 Limiters applied | 695 |

#### **Supplementary File S1: Table S3: CINAHL SEARCH Strategy**

Database:**EBSCO CINAHL Plus**

Limiters:**Published Date: 2010/01/01-2021/05/05; English language**

Search modes: Find all my search terms: **Boolean/Phrase; MeSH (MH)**

| ***ID*** | ***SEARCH QUERY*** | ***Results*** |
| --- | --- | --- |
| ***1*** | *(MH "Morals+")* | 39,454 |
| ***2*** | (MH "Psychological Distress") | 1,386 |
| ***3*** | #1 AND #2 | 77 |
| ***4*** | Moral* N2 (distress* OR suffering* OR stress* OR injur* OR concern* OR transgression* OR residue) | 1,983 |
| ***5*** | #3 OR #4 | 2,000 |
| ***6*** | (MH "Nurses+") | 236,738 |
| ***7*** | (MH "Staff Nurses") | 8,326 |
| ***8*** | (MH "Registered Nurses") | 34,831 |
| ***9*** | (MH "Students, Nursing+") | 39,895 |
| ***10*** | (MH "Advanced Practice Nurses+") | 40,216 |
| ***11*** | (MH "Practical Nurses") | 5,959 |
| ***12*** | Nurse OR Nurses | 550,168 |
| ***13*** | #6 OR #7 OR #8 OR #9 OR #10 OR #11 OR #12 | 593,823 |
| ***14*** | #5 AND #13 | 975 |
| ***15*** | **#5 AND #13 Limiters applied** | **688** |

#### **Supplementary File S1: Table S4: APA PsyInfo SEARCH Strategy**

Database: APA PsyInfo

Limiters: **Published Date: 2010/01/01-2021/05/05; English language**

Search modes: Find all my search terms: **Boolean/Phrase; MeSH (MH)**

| ***ID*** | ***SEARCH QUERY*** | ***Results*** |
| --- | --- | --- |
| ***1*** | *(MH "Morals+")* | 27,034 |
| ***2*** | (MH "Psychological Distress") | 21,029 |
| ***3*** | #1 AND #2 | 31 |
| ***4*** | Moral* adj2(distress* OR suffering* OR stress* OR injur* OR concern* OR transgression* OR residue) | 2,831 |
| ***5*** | #3 OR #4 | 2,846 |
| ***6*** | (MH "Nurses+") | 33,153 |
| ***7*** | (MH "Staff Nurses") | 870 |
| ***8*** | (MH "Registered Nurses") | 4,342 |
| ***9*** | (MH "Students, Nursing+") | 5,531 |
| ***10*** | (MH "Advanced Practice Nurses+") | 561 |
| ***11*** | (MH "Practical Nurses") | 334 |
| ***12*** | Nurse OR Nurses | 69,023 |
| ***13*** | #6 OR #7 OR #8 OR #9 OR #10 OR #11 OR #12 | 71,805 |
| ***14*** | #5 AND #13 | 377 |
| ***15*** | **#5 AND #13 Limiters applied** | **278** |

#### **Supplementary File S1: Table S5: Web of Science SEARCH Strategy**

Database: Clarivate Web of Science

Limiters: English Language; Publication date from 2010/01/01 to 2021/04/30

Search modes: Find all my search terms:

| **ID** | **SEARCH QUERY** | **Results** |
| --- | --- | --- |
| **1** | ALL FIELDS: (Morals) | 113,861 |
| **2** | ALL FIELDS: (Psychological distress) | 50,032 |
| **3** | #2 AND #1 | 318 |
| **4** | ALL FIELDS: (Moral* N2 (distress* OR suffering* OR stress* OR injur* OR concern* OR transgression* OR residue) ) | 10 |
| **5** | #4 OR #3 | 328 |
| **6** | ALL FIELDS: (Nurses) | 347,594 |
| **7** | ALL FIELDS: (Staff Nurses) | 44,930 |
| **8** | ALL FIELDS: (Registered Nurses) | 16,343 |
| **9** | ALL FIELDS: (Students, Nursing) | 42,897 |
| **10** | ALL FIELDS: (Advanced Practice Nurses) | 12,419 |
| **11** | ALL FIELDS: (Practical Nurses) | 8,471 |
| **12** | ALL FIELDS: (Nurse OR Nurses) | 365,276 |
| **13** | #12 OR #11 OR #10 OR #9 OR #8 OR #7 OR #6 | 375,485 |
| **14** | #13 AND #5 | 108 |
| **15** | **#13 AND #5 Limiters applied** | **97** |
