# Supplementary material for: Registered Nurses' and nursing students' perspectives on moral distress and its effects: A mixed‐methods systematic review and thematic synthesis
Source: Nurs Open. 2023 Jul 17;10(9):6014–32. doi: 10.1002/nop2.1913 (PMC10416007; doi:10.1002/nop2.1913)
Supplement: Supplementary file 2 — File S2. [file NOP2-10-6014-s002.docx]

## **Supplementary file S2: Summary of included studies**

| **Study author (year)**  **Country** | **Study Design** | **Data Source**  **Sample size**  **Setting** | **Aim** | **Measure** | **Analysis** | **MMAT SCORE** | **Findings** |
| --- | --- | --- | --- | --- | --- | --- | --- |
| Abdolmaleki et al. (2019)  Iran | Cross sectional survey | Emergency department nurses  N=173  Emergency departments in university hospitals | To investigate the relationship between professional independence and moral distress in nurses working in emergency departments. | Hamric et al.’s Moral Distress Scale-Revised (2012) | Descriptive and inferential statistics | 100% | **RQ1** Negative relationships were found between professional autonomy and moral distress and age and moral distress. |
| Ajoudani et al. (2019)  Iran | Cross sectional survey | Nurses  N=278  Teaching hospitals | To investigate the relationship between moral distress and burnout in Iranian nurses, as mediated by perceptions of workplace bullying. | Hamric et al.’s Moral Distress Scale-Revised (2012) | Descriptive and inferential statistics | 100% | **RQ1** Nurses’ moral distress was associated with their perceived workplace bullying. |
| Alberto Fruet et al. (2019)  Brazil | Cross-sectional survey | Nurses, nursing technicians and nursing assistants  N=46  Haematology-Oncology in a hospital | To identify the frequency and intensity of moral distress, and analyse the associations between moral distress and sociodemographic and labour characteristics. | Moral Distress Scale (MDS) - Brazilian Version | Descriptive and inferential statistics | 100% | **RQ1** Greater intensity of moral distress was connected to the denial of nurses’ advocacy role and disrespecting patient autonomy.  **RQ2B** Nurses face inconsistencies between personal and professional values that they cannot manage. As a result, they choose to leave the profession because they lack support in coping with these situations. |
| Altaker et al. (2018)  United States | Cross-sectional survey | Intensive care nurses  N=238  Hospital | To evaluate relationships among moral distress, empowerment, ethical climate, and access to palliative care in the ICU. | Hamric et al.’s Moral Distress Scale-Revised (2012) | Descriptive and inferential statistics | 100% | **RQ1** Ethical climate, access to palliative care teams, unit size, ethnicity, and type of educational degree contributed to variance in moral distress. |
| Asayesh et al. (2018)  Iran | Cross-sectional survey | Intensive care nurses  N=117  Hospital | To examine the relationship between futile care perception and moral distress. | Hamric et al.’s Moral Distress Scale-Revised (2012) | Descriptive and inferential statistics | 50% | **RQ1** There were statistically significant relationships between nurses' perceptions of severity and frequency of futile care and moral distress. Experience of ICU care correlated with moral distress. |
| Asgari et al. (2019)  Iran | Cross-sectional survey | Critical care nurses  N=142  Social security hospitals | To determine the relationship between moral distress and ethical climate with job satisfaction | Hamric et al.’s Moral Distress Scale-Revised (2012) | Descriptive and inferential statistics | 50% | **RQ1**  Nurses experienced the highest frequency of moral distress when performing unnecessary tests and treatments. Helping doctors who were not competent caused the highest-level of moral distress intensity.  **RQ2B**  Participants had a history of leaving their clinical positions. |
| Bayat et al. (2019)  Iran | Cross-sectional survey | Nurses  N= 300  Hospitals | To determine the relationship between nurses' moral distress and ethical climate | Corley et. al.’s (2001) Moral Distress Scale | Descriptive and inferential statistics | 75% | **RQ1** A statistically significant inverse relationship between hospital ethical climate and moral distress was identified. The subdomains of ethical climate (colleagues, patients, hospital, and physicians) had a statistically significant relationship with moral distress, with the exception of managers. |
| Berhie et al. (2020)  Ethiopia | Cross-sectional survey | Nurses  N= 412  Regional state referral hospital | To assess the proportion of moral distress and associated factors | Hamric et al.’s Moral Distress Scale-Revised (2012). | Descriptive and inferential statistics | 100% | **RQ1** Perceived poor communication, perceived powerlessness in decision making, inadequate staffing, and inappropriate provision of care were associated with moral distress |
| Borhani et al. (2014)  Iran | Cross-sectional survey | Nurses  N=220  Teaching hospital | To examine the relationship between moral distress, professional stress, and intent to stay in nursing. | Corley et. al.’s (2001) Moral Distress Scale | Descriptive and inferential statistics | 25% | **RQ1** Nurses had medium levels of moral distress and professional stress. Emotional involvement with patients’ problems and their relatives were important sources of stress.  **RQ2b**  The majority of nurses did not intend to leave the profession. |
| Browning (2013)  United States | Cross-sectional survey | Critical care nurses  N= 277  Critical care units | To describe the relationships between moral distress, psychological empowerment and demographics in critical care nurses caring for patients at the end of life. | Corley et. al.’s (2001) Moral Distress Scale | Descriptive and inferential statistics | 50% | **RQ1** The study confirmed a relationship between self-determination and moral distress frequency, particularly when managing difficult situations related to aggressive care and futility. Nurses who experienced greater moral distress related to deception were more likely to attach greater meaning to their work, have higher levels of autonomy and more influence at work. |
| Christodoulou-Fella et al. (2017)  Cyprus | Cross-sectional survey | Psychiatric Nurses  N= 206  Psychiatric care | To explore the frequency and intensity of morally distressing situations, the severity of symptoms of secondary traumatic stress syndrome (STSS) and the association among moral distress and STSS with sociodemographic factors and work-related features | Hamric et al.’s Moral Distress Scale-Revised (2012) | Descriptive statistics and inferential statistics | 75% | **RQ1**  Items with the highest morally distressing intensity and frequency included working with incompetent colleagues, unnecessary or unconsented treatment, suspicions of abuse, or unsafe patient-staff ratios.  **RQ2B** 20% reported intention to leave their job due to moral distress. |
| Davis et al. (2012)  United States | Cross-sectional survey | Registered nurses  N=1114  Hospital | To explore influences identified by nurses as having an impact on their ethical beliefs and whether these influences might impact levels of moral distress and the potential for conscientious objection | Hamric et al.’s Moral Distress Scale-Revised (2012). | Descriptive and inferential statistics | 50% | **RQ1**  Providing patient care that nurses did not ethically condone despite their spiritual beliefs generated moral distress.  **RQ2B**  27.7% (n=317) reported leaving a job due to moral distress. |
| Delfrate et al. (2018)  Italy | Cross-sectional survey | Registered psychiatric nurses  N=228  Hospital | To assess the presence of moral distress among mental health nurses in Italy and verify whether there is a relationship between moral distress and burnout. | Canciani et. al.’s (2016) Moral Distress Scale for Psychiatric Nurses (Italian, revised) | Descriptive and inferential statistics | 50% | **RQ1**  Insufficient material and human resources and lack of acknowledgement of nurses’ competence by other staff contributed to moral distress. |
| Dodek et al. (2019)  Canada | Cross-sectional survey | Intensive care nurses  N= 669 (428 nurses)  Intensive care units | To assess the association between moral distress and general workplace distress in ICU staff. | Hamric et al.’s Moral Distress Scale-Revised (2012) | Descriptive and inferential statistics | 25% | **RQ1** Moral distress was associated with less control over decisions and lower social support, and with increased psychological stressors and strain. |
| Dyo et al. (2016)  United States | Cross-sectional survey | Registered Nurses  N= 279  Hospital | To assess moral distress intensity and frequency in adult/ paediatric nurses in critical care and non-critical care units, and explore relationships between nurse characteristics and moral distress with intention to leave | Corley et. al.’s (2001) Moral Distress Scale | Descriptive statistics | 50% | **RQ 2B**. Moral distress frequency showed a positive relationship with intention to leave a position of employment. |
| Emmamally and Chiyangwa (2020)  South Africa | Cross-sectional survey | Critical care nurses  N=74  Private Hospital | To determine the frequency, intensity and overall severity of moral distress | Hamric et al.’s Moral Distress Scale-Revised (2012). | Descriptive and inferential statistics | 50% | **RQ1** Providing futile or excessively aggressive treatment to patients contributed most to moral distress. |
| Evanovich Zavotsky and Chan (2016)  United States | Cross-sectional survey | Emergency Department Nurses  N=198  Hospital | To examine moral distress in Emergency Department nurses and its relationship  to coping. | Hamric et al.’s Moral Distress Scale-Revised (2012). | Descriptive statistics | 50% | **RQ2B** 51% (n=101) reported they had neither considered leaving nor had left a position because of moral distress whereas 36.9% (n=73) had considered leaving and 12.1% (n=24) had left a previous position because of moral distress. 30.3% (n=60) were considering leaving their current  position because of moral distress. |
| Fard et al. (2020)  Iran | Cross sectional survey. | Nurses  N=150  Public and private hospitals | To determine the association of moral distress with sleep quality in nurses and to compare among nurses working in private and public hospitals | Corley et. al.’s (2001) Moral Distress Scale | Descriptive statistics and inferential statistics | 25% | **RQ2A** Moral distress had an impact on nurses including sleep disruption. |
| Fernandez-Parsons et al. (2013)  United States | Cross-sectional survey | Emergency Department Registered Nurses  N=51  Hospital | To explore the frequency of moral distress, intensity of moral distress, and situations that increase moral distress | Hamric et al.’s Moral Distress Scale-Revised (2012). | Descriptive and inferential statistics | 25% | **RQ1**  Following families’ wishes that were not in the best interest of the patient, unnecessary orders, prolonging death, and poor communication in the team contributed to moral distress. Unsafe staffing levels and less competent colleagues were commonly noted as causes of moral distress.  **RQ2b** Moral distress was the reason given by 6.6% of registered nurses for leaving a previous position, 20% said that they had considered leaving a position but did not, and 13.3% stated that they were considering leaving their position because of moral distress. |
| DeKeyser Ganz et al. (2013)  Israel | Cross-sectional survey | Intensive care nurses  N=291  Intensive care units | To determine levels of structural empowerment, moral distress, and the association between them. | Corley et. al.’s (2001) Moral Distress Scale | Descriptive and inferential statistics | 50% | **RQ1** There was a weak but statistically significant correlation between empowerment and moral distress. Predictors of moral distress frequency included type of unit and access to resources. Predictors of moral distress intensity included seniority and specialism type, as well as part-time status. |
| Haghighinezhad et al. (2019)  Iran | Cross-sectional survey | Intensive care nurses  N=284  Hospitals | To investigate the relationship between perceived organisational justice and moral distress | ICU Nurses’ Moral Distress Scale | Descriptive and inferential statistics | 50% | **RQ1** There was a negative relationship between nurses' perceptions of organizational justice (the fairness of procedures and efforts) and moral distress. |
| Hamaideh (2014)  N=130  Jordan | Cross-sectional survey | Mental health nurses  N=130  Hospitals and clinics | To describe the levels of moral distress experienced by Jordanian mental health nurses, to examine the relationships of moral distress with nurses’ job satisfaction, intention to leave the job, and burnout, as well as to identify the predictors of moral distress. | Moral Distress Scale for Psychiatric Nurses | Descriptive statistics | 50% | **RQ2B** Moral distress was higher among younger nurses, nurses with low-income level, nurses with less experience, nurses working in wards with higher caseloads, nurses with higher educational levels, and nurses who intended to leave their current job. |
| Harorani et al. (2019)  Iran | Cross-sectional survey | Nurses  N=300  ICU, cardiac care, and dialysis units | To determine the average frequency and intensity of moral distress, and its relationship with self-efficacy. | Corley et. al.’s (2001) Moral Distress Scale | Descriptive and inferential statistics | 25% | **RQ1** A statistically significant negative relationship between self-efficacy and moral distress was identified |
| Hatamizadeh et al. (2019)  Iran | Cross-sectional survey | Nurses  N=276  Hospital | To assess the implications of Iran’s recent health care reforms on nurses’ experience of moral distress, their perceptions of the respect for patient rights and the relationship of these variables to job and income dissatisfaction | Corley et. al.’s (2001) Moral Distress Scale | Comparison statistics | 50% | **RQ2B** Moral distress intensity and frequency was positively correlated with turnover intention. Turnover intention increased by 0.307 for each unit of moral distress intensity and 0.524 units for the moral distress frequency score. Nurses who worked in circular shifts had a score 0.188 higher than nurses with constant shifts for turnover intention. |
| Hiler et al. (2018)  United States | Cross-sectional survey | Critical care nurses  N=328  Teaching hospitals | To explore the relationships among the severity of moral distress, the practice environment, and patient safety | Hamric et al.’s Moral Distress Scale-Revised (2012). | Descriptive and predictive statistics | 50% | **RQ1** High levels of moral distress occurred when nurses deemed that the care provided was futile. The highest moral distress frequency occurred when the wishes of the patient’s family were not in the best interest of the patient. Additionally, as the practice environment deteriorated, the level of moral distress was higher. |
| Hou et al. (2021)  China | Cross-sectional survey | Emergency department nurses  N=291  Public hospitals | To explore relationships between moral distress, ethical climate, and nursing practice environment among a sample of Emergency Department nurses and determine significant predictors of moral distress in organisational environments | Sun et al.’s (2012) Chinese Version of Moral Distress Scale-Revised (MDS-R) | Descriptive and predictive statistics | 100% | **RQ1**  Poor collaboration between nurses and doctors caused moral distress. Futile care, families’ decisions that were not in the patients’ best interests, power imbalance and differing expectations of patient care also contributed to moral distress.  **RQ2B** 108 (48.21%) participants answered that they had never left or considered leaving their clinical position because of moral distress, whereas 2 (0.89%) had left a position and 114 (50.89%) had considered leaving but did not leave due to moral distress. In addition, 30 (13.45%) participants were considering leaving their current position while 193 (86.55%) were not. There were significant differences regarding the level, frequency, and intensity of moral distress among the groups that had ever left or considered leaving or were presently considering leaving their current position (all P <0.05). |
| Karanikola et al. (2014)  Italy  Aim | Cross-sectional survey | Intensive care nurses  N=556  Nursing international conference | To explore the level of moral distress and potential associations between moral distress indices and (1) nurse-physician collaboration, (2) autonomy, (3) professional satisfaction, (4) intention to resign, and (5) workload | Corley et. al.’s (2001) Moral Distress Scale | Descriptive and comparison statistics | 75% | **RQ1**  Moral distress was associated with poor collaboration between doctors and nurses, and dissatisfaction with care decisions. Moral distress was also weakly correlated with staffing ratios.  **RQ2B** The frequency of morally distressing situations was associated positively but weakly with (1) job satisfaction and (2) frequency of the intention to resign from post owing to morally distressing situations. The severity of moral distress was associated negatively, however negligibly, with work satisfaction and positively, weakly with an intention to resign from post as a result of morally distressing situations. |
| Krautscheid et al. (2020)  United States | Pilot, cross-sectional survey | Senior nursing students  N=60  University | To identify if significant associations exist between protective factors and moral distress ratings. | Wocial and Weaver’s (2013) moral distress thermometer (MDT) |  | 50% | **RQ2B**There were weak negative correlations between social support and moral distress and between protective factors and moral distress. |
| Latimer et al. (2021)  United States | Pilot, cross-sectional survey | Ventricular assistance device (VAD) coordinators (nurses)  N=36  Hospital | To explore the association between ventricular assistance device (VAD) coordinators' unique roles and responsibilities and moral distress. | Measure of Moral Distress for Health Professionals (Epstein et al., 2019) | Descriptive and inferential statistics | 25% | **RQ1**  Poor communication amongst the team and limited end of life education were highlighted added to moral distress. |
| Laurs et al. (2020)  Lithuania | Cross-sectional survey | Registered nurses  N=612  Municipal hospitals | To describe the level of moral distress experienced by nurses, situations that most often caused moral distress, and the intentions of the nurses to leave the profession. | Hamric et al.’s Moral Distress Scale-Revised (2012). | Descriptive and inferential statistics | 75% | **RQ1**  Conducting unnecessary tests, providing care that met family wishes but was not in the patients’ best interests, and not discussing prognosis with patients or families were noted as reasons for moral distress.  **RQ2B**  Nurses with high moral distress levels are more likely to consider leaving their position compared with those with moderate or low moral distress levels. |
| Moaddaby et al. (2021)  Iran | Cross-sectional survey | Intensive care nurses  N=155  Hospitals | To determine the perception of futile care and its relationship with moral distress | Corley et. al.’s (2001) Moral Distress Scale | Descriptive and inferential statistics | 100% | **RQ1** Legal and organisational considerations were statistically significant predictors of moral distress. |
| O’Connell (2015)  United States | Cross sectional survey | Critical care nurses  N=31  Internet nursing community | To explore moral distress levels in a sample of critical care nurses to determine whether gender differences exist in moral distress scores. | Hamric et al.’s Moral Distress Scale-Revised (2012 | Descriptive and inferential statistics | 50% | **RQ1** Women had statistically significantly higher moral distress compared to men |
| Pergert et al. (2019)  Sweden | Cross-sectional survey | Paediatric Nurses  N=278  Oncology department, hospital | To explore healthcare professionals’ experiences of situations that generate moral distress | Swedish Moral Distress Scale -Revised | Descriptive and inferential statistics | 75% | **RQ1** Moral distress was associated with perceptions of lack of competence and poor continuity of personnel. |
| Rathert et al. (2016)  United States | Cross-sectional survey | Trauma nurses  N=290  Trauma hospitals | To examine work environment and intrapersonal variables that may influence moral distress | Study-specific and included one item about moral distress | Descriptive and inferential statistics | 50% | **RQ1** There was a negative relationship between institutional ethics support and moral distress. A perceived lack of voice contributed to this distress. |
| Robaee et al. (2018)  Iran | Cross-sectional survey | Nurses  N=110  Hospital | To determine the level of perceived organisational support and moral distress among nurses and to investigate the relationship between these two variables. | The ICU nurses’ Moral Distress Scale | Descriptive and inferential statistics | 50% | **RQ1** There was a statistically significant relationship between work shifts and moral distress. |
| Sarkoohijabalbarezi et al. (2017)  Iran | Cross-sectional survey | Paediatric Nurses  N=120  Hospital | To investigate the relationship between professional autonomy and moral distress | Hamric et al.’s Moral Distress Scale-Revised (2012). | Descriptive and inferential statistics | 50% | **RQ1** There was a significant positive relationship between moral distress and professional autonomy. |
| Soleimani et al. (2019)  Iran | Cross-sectional survey | Nurses  N=193  Hospital | To examine the relationship between spiritual well-being and moral distress among a sample of nurses and to study the determinant factors of moral distress and spiritual well-being in nurses. | Hamric et al.’s Moral Distress Scale-Revised (2012) | Descriptive and inferential statistics | 75% | **RQ1** Gender and education were independent predictors for moral distress. Factors that became significant following adjustment of other factors for moral distress were shift rotation, tendency to leave jobs, and age. |
| Trautmann et al. (2015)  United States | Cross-sectional survey | Emergency department nurse practitioners  N=207  Hospital | To investigate moral distress among emergency department nursing practitioners and to examine relationships between moral distress, level of practice independence, and intent to leave their position. | Hamric et al.’s Moral Distress Scale-Revised (2012). | Descriptive and inferential statistics | 25% | **RQ1**  Moral distress was associated with poor patient care, inadequate staff communication, and working with incompetent colleagues.  **RQ2B**  Approximately 25% of participants stated they left a position because of moral distress. |
| Wands (2018)  United States | Cross-sectional survey | Registered nurse anaesthetists  N=134  Hospital | To examine the relationship between moral distress, patient safety, and intensive care nurses ethical decision making skills. | Ethical Stress Scale (ESS) | Descriptive and inferential statistics | 25% | **RQ1** There was a significant relationship between moral distress and perceived ethical assessment abilities. |
| Wilson et al. (2013)  United States | Cross-sectional survey | Critical care nurses  N=105  Hospital | To examine the level and frequency of moral distress in staff nurses working in two units in the critical care division of one hospital and to gather information to potentially guide future support, resources and interventions for moral distress in staff nurses. | Corley et. al.’s (2001) Moral Distress Scale | Descriptive and inferential statistics | 25% | **RQ1**  Futile and unnecessary care contributed to moral distress.  **RQ2B**  A high percentage of the nurses surveyed reported having left or considered leaving a position because of morally distressing situations. |
| Woods et al. (2015)  New Zealand | Cross-sectional survey | Registered nurses  N=412  Hospital | To determine the frequency and intensity of moral distress experienced by registered nurses in New Zealand and to examine whether or not this distress led to nurses contemplating leaving their current positions | Hamric et al.’s Moral Distress Scale-Revised (2012). | Descriptive and inferential statistics | 50% | **RQ1**  Management decisions causing suboptimal care, patient care suffering, and working with less competent colleagues caused distress. Poor communication and patient suffering due to lack of care continuity also contributed to moral distress.  **RQ2B**  48% reported having considered leaving their position due to moral distress. |
| Yeganeh et al. (2019)  Iran | Cross-sectional survey | Intensive care nurses  N=180  Hospitals | To determine the relationship between professional autonomy and moral distress of ICU nurses. | Corley et. al.’s (2001) Moral Distress Scale | Descriptive and inferential statistics | 50% | **RQ1**  There was a positive and significant relationship between moral distress and total professional autonomy scores. |

## **Qualitative studies**

| **Study author and year**  **Country** | **Study Design** | **Data Source**  **Sample size**  **Setting** | **Aim** | **Data Collection** | **Data analysis** | **MMAT** | **Findings relevant to review question** |
| --- | --- | --- | --- | --- | --- | --- | --- |
| Atashzadeh et al. (2012)  Iran | Qualitative descriptive research | Intensive care nurses and educators  N=31  Hospital | To explore moral distress among ICU nurses in Iran | Individual interviews | Content analysis. | 75% | **RQ1** Moral distress was caused by institutional barriers and constraints, difficulties in communicating with patients, colleagues, and families, futile care or errors in care, and poor allocation of resources or responsibilities. |
| Caram et al. (2019)  Brazil | Qualitative descriptive research | Intensive care and surgical nurses  N=13  Intensive care and surgical units | To analyse how nurses reach for the telos in their practice within the context of moral distress | Observation and semi-structured interviews | Thematic analysis | 100% | **RQ1** Contradictions in work can lead to nurses feeling unable to express nursing virtues in their clinical practice. Managing economic and political priorities in determining care contributed to moral distress. |
| Deady and McCarthy (2010)  Ireland | Qualitative descriptive research | Registered psychiatric nurses  N=8  Acute psychiatric wards | To explore psychiatric nurses’ experiences of moral distress within acute care settings. | Individual interviews | Thematic analysis | 100% | **RQ1**  Moral distress occurred when people had difficulty sharing their professional views on decisions they disagreed with. This was more difficult when they believed colleagues did not value their views or senior colleagues would not act upon their concerns. This also occurred when they believed that professional conflict and/or potential legal issues took precedent over patient care, or the standard of care was below their own sense of best practice.  **RQ2A**  Participants commonly felt self-doubt, guilt, and frustration when experiencing moral distress.  **RQ2B**  Some participants referred to moving job as a coping strategy. |
| Crespo Drago et al. (2020)  Brazil | Qualitative descriptive research | Nurse managers  N= 17  Hospital | To describe situations and elements involved in the moral distress process | Individual interviews and comic book completion | Discursive textual analysis | 100% | **RQ1** Nurse managers reported that conflicts in the organisation and the team, poor working conditions, lack of autonomy in managing people, and the impact of working as a manager contributed to moral distress. |
| de Sousa Vilela et al. (2021)  Brazil | Qualitative descriptive research | Intensive care nurses  N=12  Hospital: intensive care | To understand ethics and moral distress expressions in intensive care nursing practice. | Individual interviews | Thematic content analysis | 50% | **RQ1** Nurses experienced moral distress when faced with situations with which they did not agree and could not change and the inability to enact patient advocacy. |
| Escolar Chua and Magpantay (2019)  Philippines | Qualitative descriptive research | Senior nursing students  N=14  University | To explore the moral distress and ethical concerns encountered by undergraduate nursing students. | Individual interviews | Thematic analysis | 75% | **RQ1** Three themes were identified. Themes described how unprofessional behaviour from other healthcare workers, a sense of powerlessness to change wider issues, and differing expectations on health between the students and the community they cared for contributed to moral distress. |
| Forozeiya et al. (2019)  Canada | Qualitative descriptive research | Intensive care nurses  N=7  Hospital: Intensive care | To describe intensive care nurses’ experiences of coping with moral distress. | Individual interviews | Thematic analysis | 100% | **RQ1**  Moral distress was related to a lack of clarity around patients’ wishes, others’ decisions, and the amount of information provided to families. Another factor that contributed to moral distress was the impact of poor management of end-of-life care.  **RQ2A**Nurses felt traumatised, angry, stressed and/or frustrated. The social consequences were that they withdrew from friends and family, and discussed dread of going to work.  **RQ2B** Nurses contemplated working fewer hours or ending their employment. |
| Jansen et al. (2020)  Norway | Qualitative descriptive research | Registered Psychiatric nurse specialists  N=16  Hospitals | To describe sources of moral distress and what characterises moral distress in acute mental care nursing settings. | Individual interviews | Thematic analysis | 75% | **RQ1**  Moral distress occurred when nurses felt they had failed their patients; had insufficient time for therapeutic work; contributed to coercive measures in patient care, and refusals to limit patients’ autonomy.  **RQ2A** The effects moral distress were evident in professional and personal lives, with feelings of frustration, guilt, sadness, inadequacy, and doubt. Some participants doubted their own actions or found less meaning in life/work. When not working, they withdrew from social contact. Poor sleep and high blood pressure were experienced.  **RQ2B**  Several participants contemplated leaving their jobs. |
| Maluwa et al. (2012)  Malawi | Qualitative descriptive research | Nurses  N=20  Various settings in one region of Malawi | To explore the existence of moral distress. | Individual interviews | Thematic analysis | 75% | **RQ1** Moral distress was mainly caused by difficulties associated with patient care, colleagues and administration, especially higher authorities. |
| Pavlish et al. (2016)  United States | Qualitative descriptive research | Nurse leaders  N=100  Association of nurse leaders | To explore nurse leaders' experiences working in ethically difficult situations and helping nurses cope with moral distress. | Critical incident narratives | Descriptive analysis | 25% | **RQ1** Poor work environment, power dynamics and a culture of silence contributed to moral distress. Strained relationships between team members and a lack of awareness of ethics and ethics resources were also factors in moral distress. End-of-life issues were also relevant in moral distress. |
| Prestia et al. (2017)  United States | Qualitative exploratory study | Chief Nursing Officers  N=20  Various states across the United States | To explore if moral distress and its lingering residue were experienced by Chief Nursing Officers. | Individual interviews | Content analysis | 50% | **RQ1** Moral distress was described as related to issues of salary and staff compensation, financial constraints, managing nurse-to-patient ratios, counter-productive relationships, and authoritative improprieties.  **RQ2A** The effects were feeling isolated, powerless, psychologically unsafe, struggling with moral compasses, moral residue, and the need to gather strength from networking. |
| Renno et al. (2018)  Brazil | Qualitative descriptive research | Undergraduate nursing students  N=58  University | To identify the existence of moral distress caused by ethical conflict and dilemmas experienced during their nursing education. | Focus group interviews | Thematic content analysis | 50% | **RQ1** Teachers’ behaviour was sometimes a source of moral distress. Additionally, feeling powerless to resolve ethical dilemmas in health services, such as hierarchy, holding precarious roles, or seeing health professionals using outdated knowledge also contributed to moral distress. |
| Rezaee et al. (2019)  Iran | Qualitative descriptive research | Oncology nurses  N=25  Hospitals | To explain nurses' perceptions of ethical challenges in caring for cancer patients in Iran. | Individual interviews | Content analysis | 100% | **RQ1** The two factors that caused moral distress were poor communication with patients and families, and provision of futile care that caused suffering to patients. |
| Ritchie et al. (2018)  Canada | Qualitative descriptive research | Nurse practitioners  N=6  Urban setting in Western Canada | To understand the experience of moral distress for nurse practitioners in continuing care, exploring similarities and variances in their experiences compared with the general nursing population. | Individual interviews | Interpretive description | 50% | **RQ1** Moral distress were caused by the inability to provide good care because of outside constraints, and the impact of perceptions of their roles as nurse practitioners. Power struggles between participants and doctors, institutional policies and expectations colliding with realities of practice, and struggling to meet individuals’ demands contributed to moral distress. |
| Silverman et al (2021)  United States | Qualitative descriptive research | Acute care nurses  N=31  Hospital | To explore causes of moral distress in nurses caring for Covid-19 patients and identify strategies to enhance their moral resilience. | Individual interviews and focus groups | Qualitative content analysis | 75% | **RQ1** Causes of moral distress in participants included a lack of knowledge on how to treat Covid-19, feeling overwhelmed by Covid-19, a fear of being exposed to the virus and providing suboptimal care as a result. Other causes of moral distress included intra-professional tension or poor communication, the impact of policies that prevented them from taking on their role as a nurse, a lack of resources, and practising within crisis standards of care. |
| Wiegand and Funk (2012)  United States | Qualitative descriptive study | Critical care nurses  N=47  Hospital | To determine clinical situations that caused critical care nurses to experience moral distress. | Open-ended survey | Thematic analysis | 25% | **RQ1** End-of-life issues such as futile care or analgesic medication difficulties caused moral distress. Feeling unheard or providing a lower standard of care were also issues, as well as conflicts with families and colleagues. **RQ2A** Reduced morale, helplessness, exhaustion, depression and anger.  **RQ2B** Reduced job satisfaction was also a consequence of moral distress. |
| Wolf et al. (2016)  United States | Qualitative exploratory research | Emergency department nurses  N=17  Conference | To explore the nature of moral distress as it is experienced and described by emergency nurses | Focus groups | Constant comparison | 100% | **RQ1**  Use of technology was identified as a persistent challenge. Patient care was compromised by the requirement for excessive documentation, lack of time, resources, unreliable technology, inadequate staffing and administrative decisions. Power imbalance and role conflicts arose from differing expectations between nurses, physicians, and hospital administrators.  **RQ2A** This resulted in feeling powerless, guilty, angry, fearful and frustrated. Physical issues such as fatigue, sleep difficulty, digestive issues, appetite change, and high blood pressure impacted nurses’ health. Use of unhelpful coping strategies such as food or alcohol also impacted health. |
| Woods (2020)  New Zealand | Qualitative descriptive research | Nurses  N=140  Hospital | To discuss causes and effects of moral distress. | Qualitative survey | Thematic analysis | 75% | **RQ2A**Unsupportive “system” of institutions, difficulties with managers, bullying or poor practice from colleagues, and the effect of moral residue (including guilt and doubt) contributed to moral distress.  **RQ2B** Nurses were struggling under an increasing weight of moral residue to maintain their ethical standards within an increasingly difficult ethical climate. |
| Harrowing and Mill (2010)  Uganda | Ethnographic study | Nurses  N=24  Referral centre | To describe the manifestation and impact of moral distress as it was experienced by Ugandan nurses who provided care to HIV infected or affected people. | Ethnographic methods | Thematic analysis | 75% | **RQ1**  Moral distress occurred when a lack of resources put patients’ at risk.  **RQ2A**Nurses experiencing moral distress experienced inadequacy, helplessness, and hopelessness. They felt frustrated, traumatised and at risk of burnout. They also experienced fatigue, despair, and negative attitudes to work. |
| Hsun-Kuei et al. (2018)  Taiwan | Grounded theory | Staff nurses  N=25  Teaching hospitals | To reconstruct the model of moral distress using grounded theory. | Interviews | Constant comparison | 75% | **RQ1**  Moral distress was caused by co-workers, the wider system, and Chinese culture. Systemic barriers such as nursing shortage and medical resources waste also contributed to moral distress.  **RQ2A** Nurses felt frustrated and angry. |
| Musto and Schreiber (2012)  Canada | Grounded Theory | Registered nurses and registered psychiatric nurses  N=12  Inpatient and community care | To develop a substantive theory of the processes mental health nurses use when they experience moral distress. | Individual interviews | Constant comparison | 75% | **RQ1**  Incidents that effected nurses’ abilities to maintain patient safety caused moral distress, along with feeling they had not lived up to the requirements of the nurse-patient relationship.  **RQ2A**  Moral distress caused self-doubt regarding nursing judgements, frustration, anger and powerlessness. |
| Porr et al. (2019)  Canada | Grounded theory | Registered nurses  N=24  Community | To uncover the process of behaviours enacted by community nurses when experiencing ethical conflicts. | Individual interviews | Constant comparison | 75% | **RQ1**  The main source of moral distress was an inability to deliver quality care. Nurses experienced moral distress when struggling with moral dilemmas and conflict.  **RQ2A**Nurses experienced moral residue, or lingering distress, when conflicts that caused moral distress were not fully resolved. Moral conflict resulted in frustration, doubt, guilt, and anxiety. Moral residue was an issue for participants. |
| Reader (2015)  United States | Narrative research | Nursing students  N=15  University | To describe experiences of moral distress among students enrolled in  associate degree nursing programs. | Individual interviews | Thematic: across-case analysis and narrative: within-case analysis | 25% | **RQ1** Moral distress was experienced as a result of the negative behaviours displayed by professional staff and nursing faculty members. |
| Wojtowicz et al. (2014)  Canada | Naturalistic Enquiry | Nursing students  N=7  University | To explore nursing students’ experiences of moral distress during clinical rotations on an inpatient psychiatric unit. | Individual interviews | Inductive thematic analysis | 75% | **RQ1** The experience of nursing not living up to previous ideals, hierarchies that left participants feeling powerless, misleading patients or withholding information, and feeling a lack of support about concerns led to moral distress. |
| De Brasi et al. (2021)  Italy | Phenomenology | Onco-haematology nurses  N=28  Hospital | To explore the causes of morally distressing events, feelings experienced by nurses and coping strategies utilised. | Individual interviews | Interpretative phenomenological analysis | 100% | **RQ1** Poor communication with patients, relatives, or healthcare professionals contributed to moral distress. Being unable to meet patients’ wishes in end-of-life care because of family attitudes or feeling forced to conceal the truth from their patients also contributed to moral distress. The participants also identified causes of moral distress found in other studies such as working with dangerous staffing levels, doing tasks that they did not feel competent to complete, and providing care that did not relieve patients’ suffering. |
| Choe et al. (2015)  South Korea | Phenomenology | Critical care nurses  N= 14  Hospital critical care setting | To understand moral distress from the perspective and experience of critical care nurses. | In-depth, individual interviews | Phenomenological using Giorgi's (2009) approach | 100% | **RQ1** Unnecessary medical treatments or care that did not allow patients dignity caused moral distress. Observing colleagues (both nursing and medical) who engaged in or covered up unethical practice or lacking autonomy in decision-making over treatment options also contributed to moral distress. Moral distress was also generated by conflicts with institutional policy. |
| Ko et al. (2019)  Taiwan | Phenomenology | Nurses  N= 32  Hospital | To analyse the main causes of moral distress. | Individual interviews | Interpretative interactionist analysis | **25%** | **RQ1** Moral distress occurred when patients were not aware of diagnoses, medical decisions did not appear to be optimal for patients, and when patients were not afforded a good death. A lack of confidence, power hierarchies in teams, having strong intentions for good to support patients, and the wider Oriental culture were deemed to exacerbate moral distress. |
| Nikbakht Nasrabadi et al. (2018)  Iran | Phenomenology | Nurse managers  N=14  Hospital | To investigate the lived experiences of clinical nurse managers regarding moral distress. | Individual interviews | Thematic analysis | 75% | **RQ2a**  Moral distress caused a sense of betraying moral principles and caused confusion, anxiety, and concern. Other outcomes of moral distress noted were self-blame and psychological suffering. The feeling that institutional commitments took priority over individuals’ obligations contributed to moral distress.  **RQ2B** Moral distress led to leaving managerial positions and resignation. |
| Robinson and Stinson (2016)  United States | Phenomenology | Emergency department nurses  N=8  Hospital | To determine how emergency nurses define moral distress, experience moral distress and its effectst, and identify strategies to cope. | Individual interviews | Thematic analysis | 50% | **RQ1** Participants discussed causes of moral distress including differences in patient care depending on whether the family was present, regret for care they felt forced to provide or guilt about care they gave to patients they preferred not to care for. |

## *Mixed methods studies*

| **Study author and year**  **Country** | **Design** | **Setting**  **Participant characteristics** | **Aim** | **Data collection** | **Data analysis** | **MMAT** | **Findings relevant to review question** |
| --- | --- | --- | --- | --- | --- | --- | --- |
| Langley et al. (2015)  South Africa | Mixed-methods | Intensive care nurses  *N*=65  Hospital | To explore and describe nurses’ experiences of situations that involve end-of-life care and evoke moral distress, the consequences of these situations and the means used to manage distress. | Bespoke survey instrument  Focus groups (*n=*4) | Descriptive statistics  Content analysis | 25% | **RQ1**  Five categories covered nurses’ experiences of moral distress: lack of experience or competence by colleagues, a limit on resources available, poor consultation or communication, issues around end of life such as futile care or choosing to withdraw treatment, and a lack of support. |
| Mehlis et al. (2018)  Germany | Prospective mixed-methods | Oncologists and oncology nurses  *N*=89 (50 nurses)  Hospital | To examine moral distress related to end-of-life decision making in oncologists and oncology nurses. | Survey  Moral Distress Thermometer validated German version  Open-ended question on causes of moral distress | Descriptive and inferential statistics  Content analysis | 25% | **RQ1**  Treatment limitation for patients with advanced cancer, patients’ suffering, and uncertainty about ethical issues contributed to moral distress. |
| Krautscheid et al. (2017)  United States | Mixed-methods | Senior nursing students  *N*=267  University | To assess moral distress among nursing students, describe ethical dilemmas contributing to moral distress in practice settings, and identify reasons for inaction when encountering dilemmas. | Survey  Demographic data  Wocial and Weaver’s (2013) Moral Distress Thermometer  A brief written narrative describing clinical situations contributing to moral distress and reasons for not taking action during distressing situations | Descriptive statistics  Qualitative content analysis | 75% | **RQ1**  Students reported causes of moral distress included witnessing compromised best practice in care, a lack of respect for patients’ dignity, feeling powerless to speak up about issues, and navigating differences between their own and their patients’ values. |
| Prompahakul et al. (2021)  Thailand | Mixed methods | General Nurses  *N*=472 (survey) *N=*20 (Interviews)  Hospital | To describe the experience of moral distress and related factors | Survey  Epstein et al.’s (2019). Measure of Moral Distress for Healthcare Professionals (MMD-HP)  Interviews | Descriptive and inferential statistics  Thematic analysis | 75% | **RQ1** Factors that caused moral distress included challenges providing end-of-life care, systems-level issues, work units and prior experiences of moral distress.  Feeling powerless, end-of-life issues, and difficulties in teams (such as poor communication, incompetence or unacceptable behaviour of colleagues) prompted moral distress. |
| Sauerland et al. (2014)  United States | Mixed methods survey | Acute and critical care registered nurses  *N*=225  Hospital | To explore moral distress, moral residue, and perception of ethical climate among registered nurses in an academic medical centre | Corley et. al.’s (2001) Moral Distress Scale  Olson’s (1998) Hospital Ethical Climate Survey and two open ended questions | Descriptive statistics  Thematic analysis | 25% | **RQ1** Not having time and resources to provide optimal patient care contributed to moral distress, as did paperwork and policies that caused them to perceive that patients’ rights came second to institutional policies. The effect of colleagues who were poorly trained, bullies, or unethical also contributed to moral distress.  **RQ2A** Psychological distress, loss of confidence and a sense of being unsupported. Physical expressions of stress such as alopecia. |
| Varcoe et al. (2012)  Canada | Mixed methods | Nurses  *N*=292  Hospital | To identify how nurses experience  and respond to morally distressing experiences or the effects of moral distress on patient  care. | Survey instrument:  Corley et. al.’s (2001) Moral Distress Scale  Including open-ended questions | Descriptive statistics  Interpretive descriptive analysis | 50% | **RQ1**Systemic issues such as overwork or incompetence contributed to moral distress. Patient suffering or being judged also caused distress.  **RQ2A**Nurses felt haunted, shocked, anxious, and drained emotionally. They expressed anger and frustration toward institutional situational constraints of workload and their position in the healthcare hierarchy.  **RQ2B** Twenty participants reported having left their units or positions or opting for early retirement. 13 planned to leave through early retirement, long-term disability, or transferring to other units. In contrast, a few participants asserted that the moral distress they described 'motivated me' and 'increased my resolve'. |
